# Inflammatory bowel disease activity threatens ankylosing spondylitis: implications from Mendelian randomization combined with transcriptome analysis

**DOI:** 10.3389/fimmu.2024.1289049

**Published:** 2024-02-28

**Authors:** Yimei Ding, Jiaxu Chen, Rouxin Li, Luan Xue

**Affiliations:** Department of Rheumatology and Immunology, Yueyang Hospital of Integrated Traditional Chinese and Western Medicine, Shanghai University of Traditional Chinese Medicine, Shanghai, China

**Keywords:** inflammatory bowel disease, ankylosing spondylitis, Mendelian randomization, disease activity, transcriptome analysis

## Abstract

**Background:**

Inflammatory bowel disease (IBD) and ankylosing spondylitis (AS) share common traits of chronic recurrent inflammation affecting both the intestines and joints. Epidemiological studies have revealed that the incidence of AS has jumped from 0.3% to 3% among patients with IBD. However, these findings do not definitively establish a causal relationship whereby IBD directly leads to the development of AS. Moreover, whether the activity of IBD will have an impact on this process remains a pending question.

**Methods:**

Two-sample Mendelian randomization (MR) analyses were employed across multiple datasets to investigate the potential of IBD as a risk factor for AS. The pathogenic genes of AS were identified by MR analysis of expression quantitative trait locus. Risk scores for active and inactive patients were calculated by single-sample gene set enrichment analysis. Comparative assessments encompassing alterations in risk transcription factor activity, shifts in signaling pathways, and variances in immune cell profiles were conducted between active and inactive patients. Moreover, the correlation of immune cells and risk genes was quantified.

**Results:**

A total of 6 MR analyses, conducted across 3 exposure datasets and 2 outcome datasets, consistently revealed that IBD substantially elevates the risk of AS development. The MR analysis of the two outcome datasets identified 66 and 54 risk genes, respectively. Notably, both the risk scores computed from the two distinct sets of risk genes were notably higher in active patients compared to their inactive counterparts. Discernible variations in the activity of risk-associated transcription factors were observed between active and inactive patients. In addition, three inflammatory pathways exhibited marked activation in active patients. Moreover, seven specific immune cell types, closely linked to disease activity, exhibited statistically significant correlations with the identified risk genes.

**Conclusion:**

By combining Mendelian randomization with transcriptome analysis, this study postulates IBD as a significant risk factor for AS, and further presents innovative evidence for the impact of IBD activity on the progression of AS.

## 1 Introduction

Inflammatory bowel disease (IBD), including Crohn’s disease (CD) and ulcerative colitis (UC), is a chronic immune-mediated disorder that affects the gastrointestinal system (1). Ankylosing spondylitis (AS), also termed as radiographic axial spondyloarthritis, is a chronic inflammatory disease affecting the joints and spine (2). Both IBD and AS are characterized by recurrent inflammation with intestinal and joint involvement (3, 4). A substantial body of evidence from clinical manifestations, epidemiology, and pathogenesis has collectively substantiated their interconnectedness. Arthritis and spondyloarthropathy stand as classic extraintestinal manifestations of IBD, while IBD also constitutes a prevalent extra-articular feature of AS (5). Inflammatory bowel disease arthritis and AS belong to the broader concept of spondyloarthritis (3). The concurrent effectiveness of TNF-α inhibitors in both diseases also hints at their shared underlying mechanisms (6, 7). Particularly noteworthy is the fact that while the incidence of AS ranges from 0.09% to 0.3% in healthy populations, its prevalence surges to approximately 3% (95% CI 2-4%) among IBD patients, marking a more than tenfold increase (8, 9).

However, little is known about the intricate mechanism underpinning the connection between IBD and AS. Moreover, the observed epidemiological associations, reflecting an elevated AS incidence within the IBD population, were limited, leaving us with a compelling query: Does IBD function as a causative risk factor, driving the increased incidence of AS? Alternatively, could these conditions share upstream co-pathogenic elements that predispose them to co-occurrence, lacking a direct causal link? Furthermore, if IBD does indeed contribute to the development of AS, would the activity of IBD have an impact on this process? Given the recurrent and relatively low-incidence nature of IBD and AS, conventional clinical investigations face challenges in addressing these inquiries effectively.

Mendelian randomization (MR) analysis utilizes genetic variants as instrumental variables to simulate natural experiments, enabling the exploration of causal relationships between exposures and outcomes (10). This study uniquely employed a combination of MR analysis and transcriptome analysis to investigate the effect of gene expression levels on disease risk, providing a novel molecular-level approach for unraveling these complex clinical inquiries.

## 2 Materials and methods

**Figure 1:**
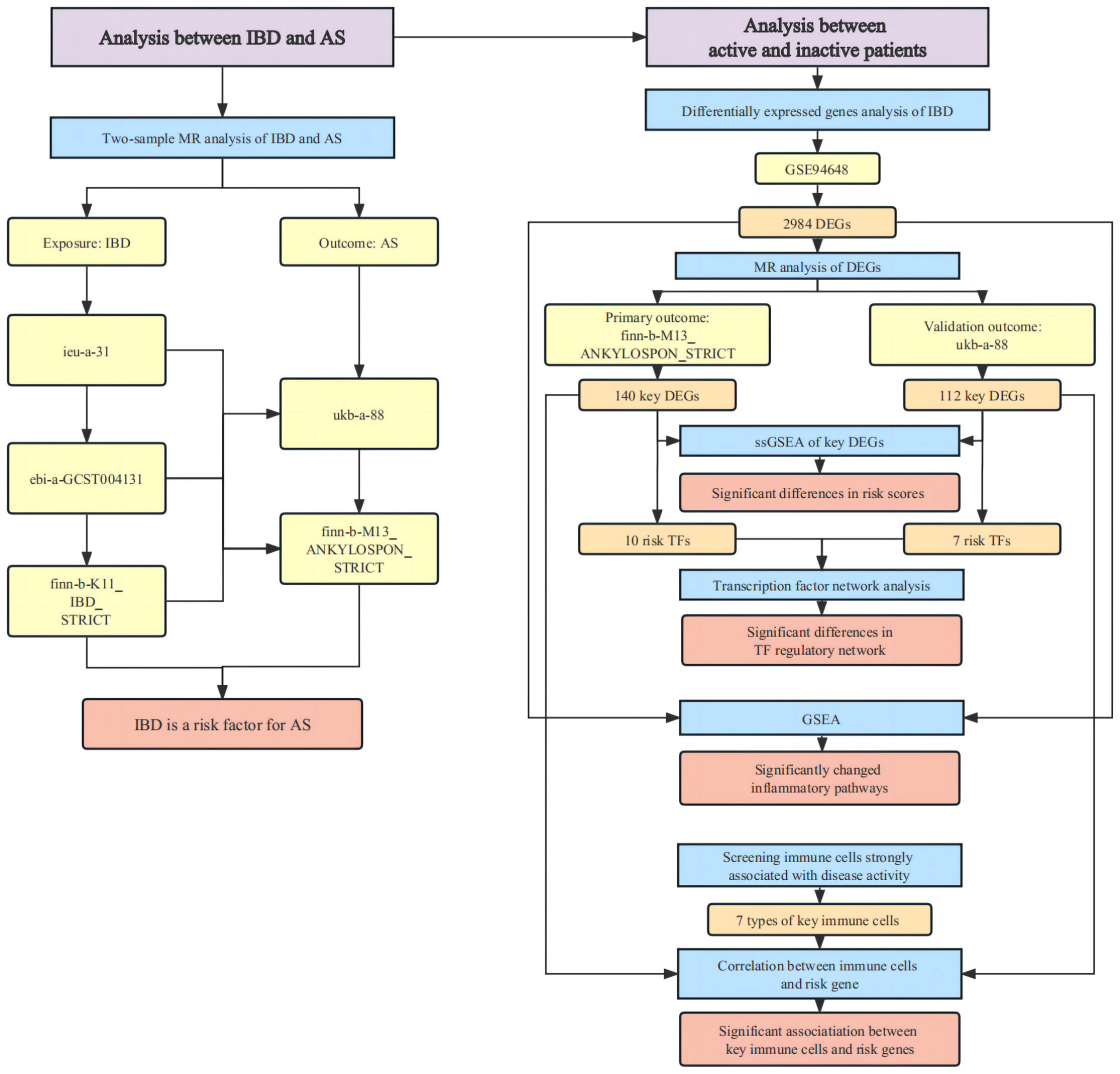
Flow chat of the overall design.

The overall design of the study were depicted in **Figure 1**.

### 2.1 Mendelian randomization analysis

The genome-wide association studies (GWAS) data of IBD and AS was acquired from IEU OpenGWAS project (https://gwas.mrcieu.ac.uk/). To ensure result validation, various datasets from distinct consortiums meeting a threshold of over 9,000,000 SNPs were employed as both exposures and outcomes. MR Analysis was performed separately across each exposure and outcome dataset. The selection criteria for instrumental variable (IV) were as follows: (1)The IV was associated with the risk factor. (2) The IV was not associated with confounders. (3) The IV influenced outcome only through the risk factor. The selection of IV was based on a threshold of P< 5x10^-8^ and r = 0.001. However if fewer than 20 IVs were available, the threshold was adjusted to P< 5x10^-6 and r = 0.01 (11). SNPs that were directly related to the outcome (P_outcome<_ 5x10^-6^) were removed first. The F statistic was used to evaluate weak instrumental variables and was calculated by the following formula: $F={\left(\frac{\beta_{x}}{se(\beta_{x})}\right)}^{2}$ (12). IVs with F<10 were excluded. Then, the Phenoscanner database (http://www.phenoscanner.medschl.cam.ac.uk/) was used to screen out IVs that could potentially be associated with confounding factors or strongly correlated with outcomes. The confounding factors included in this study referred to the possible pathogenic factors, which mainly encompassed mechanical stress, infections and psoriasis (2). The degree of heterogeneity was assessed using Cochran’s Q statistic. To detect horizontal pleiotropy within inferred causal relationships from MR analysis, the MR-PRESSO test and MR Egger were employed. The outcome from the inverse-variance weighted (IVW) regression model was adopted as the primary result, with significance set at P-value< 0.05. The leave-one-out analysis was performed to illustrate the individual impact of each SNP on the overall outcomes. R packages “TwoSampleMR” (https://mrcieu.github.io/TwoSampleMR/) and “MR-PRESSO” (https://github.com/rondolab/MR-PRESSO) were applied for MR analysis (13, 14).

### 2.2 Identification of key genes in IBD leading to AS

Gene expressions were obtained from Gene Expression Omnibus (GEO) database (http://www.ncbi.nlm.nih.gov/geo/) under the accession number GSE94648 (15). The “Lmfit” function within the “limma” package (http://bioconductor.org/packages/release/bioc/html/limma.html) was used to fit linear model for each gene in the given array series (16). The function “contrasts.fit” was used to calculate estimated coefficients and standard errors. Genes with adjusted P-values< 0.05 were identified as differentially expressed genes (DEGs) in IBD. The corresponding expression quantitative trait locus (eQTLs) were obtained from eQTLGen database (https://eqtlgen.org). Each eQTL was treated as an exposure, and a rigorously defined AS GWAS dataset (finn-b-M13_ANKYLOSPON_STRICT) was selected as the primary outcome, while another dataset, ukb-a-88, was chosen as the validation outcome. The criteria for IVs included meeting the threshold of P< 5x10^-8 and r = 0.001. If only a single SNP was available, the Wald ratio was utilized as the primary result. With a binary outcome and a dichotomous IV, log risk ratio estimate (dichotomous IV) = $\frac{{\bar{y}}_{1}-{\bar{y}}_{0.}}{{\bar{x}}_{1}-{\bar{x}}_{0}}$ , where ${\bar{y}}_{j}$ is commonly the log of the probability of an event, or the log odds of an event, in genetic subgroup j (17). In cases of multiple SNPs, the IVW regression model was adopted as the primary result. The ${\hat{\beta }}_{IVW}$ was estimated by ${\hat{\beta }}_{IVW}=\frac{\sum_{k}{\hat{\beta }}_{Xk}{\hat{\beta }}_{Yk}\sigma_{\bar{Y}k}^{2}}{\sum_{k}{\hat{\beta }}_{Xk}\sigma_{\bar{Y}k}^{2}}$ (17).

Significance in the main result was determined by a P-value< 0.05. Considering that this study focused on changes in general rather than specific genes, the original P-value rather than the adjusted P-value was chosen as a screening criterion to avoid excessive false negative rates. The remaining procedures of MR analysis were consistent with those detailed in the previous section. Genes corresponding to eQTLs derived from MR analysis were identified as key genes within IBD contributing to the development of AS. The protein quantitative trait loci (pQTL) data were derived from a large scale studies on circulating plasma proteins and genetic associations from deCODE, which provided data of 4,907 proteins (18). The causal relationship between protein expression and AS risk was further verified by MR analysis with pQTL data. The parameters of pQTL analysis were consistent with those of eQTL.

### 2.3 Calculation of risk score

Genes were categorized based on the odds ratio (OR) determined by MR analysis. Those with an OR greater than 1 were designated as risk genes, while those with an OR less than 1 were categorized as protective genes. Subsequently, utilizing the lists of risk and protective genes, the enrichment score was computed through single-sample gene set enrichment analysis (ssGSEA) to generate the risk and protective scores (19). The ssGSEA method started by rank-normalizing all the genes in a given sample in order of the expression, from largest to smallest. Enrichment scores were then generated based on the Empirical Cumulative Distribution Functions (ECDF) of the given gene set (in this study, the risk gene set of AS) and the remaining genes. The enrichment score was obtained by an integration of the difference between the ECDFs. This algorithm was executed using the R package “GSVA” (http://bioconductor.org/packages/release/bioc/html/GSVA.html). The comparison of scores between the two groups was conducted employing the Wilcoxon test.

### 2.4 Transcription factor analysis

The R package “RTN” was imported to analyze the transcription factors (TFs) in risk genes (20). This package offers a systematic approach to establish Transcriptional Network Inference (TNI) through a sequence of three steps: (i) computing mutual information between a regulator and all potential targets, and removing non-significant associations by permutation analysis; (ii) removing unstable interactions by bootstrapping; and (iii) applying the algorithm for the reconstruction of accurate cellular networks. The set of genes controlled by a given TF forms a regulon. The activity score of regulon corresponding to each transcription factor was determined using TNI and presented in a heatmap. One-tailed gene set enrichment analysis was utilized to ascertain the potential association of the regulon with a specific phenotype. In parallel, a two-tailed GSEA was employed to investigate whether the regulon demonstrates either a positive or negative correlation with the phenotype.

### 2.5 Gene set enrichment analysis

Gene set enrichment analysis (GSEA) was used to examine and clarify coordinated alterations at the pathway level across distinct phenotypes (21). The analysis centered on discerning pathway variations between active patients and inactive patients.

### 2.6 Immune cells analysis

The “xCell” package (https://github.com/dviraran/xCell), built on a spillover compensation technique, offered methodologies to predict 64 immune and stromal cell types with precision (22). This approach involved input of a gene expression dataset (such as FPKM or TPM) normalized to gene length. The ssGSEA scores were calculated for each of the 489 gene signatures and the scores of all signatures corresponding to a cell type were then averaged. Then the spillover compensation was performed for each row using linear least squares. All the values were then combined to create the final xCell score. Using this package, enrichment scores for 34 distinct immune cell types were computed. Univariate regression analysis and least absolute shrinkage and selection operator (LASSO) regression analysis were conducted on these 34 immune cell types, aiming to identify those most strongly linked to disease activity. The “cor” function within the “stats” package was employed to assess the correlation between risk genes and immune cell populations.

## 3 Result

### 3.1 MR analysis: IBD as a risk factor for AS

**Figure 2:**
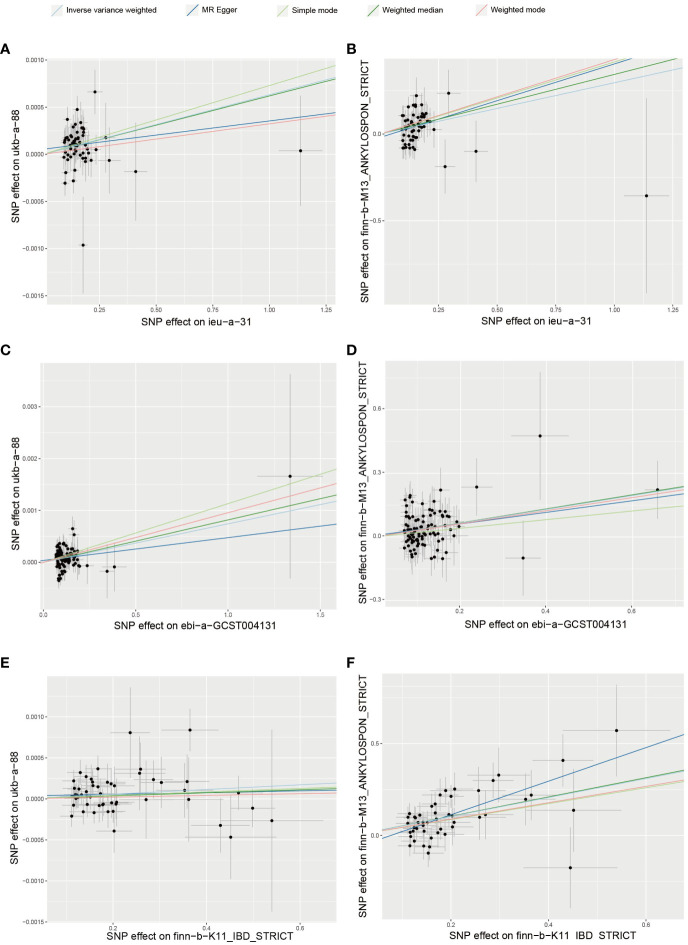
Mendelian randomization analyses between IBD and AS **(A)** exposure:ieu-a-31; outcome: ukb-a-88 **(B)** exposure: ieu-a-31; outcome: finn-b-M13_ANKYLOSPON_STRICT **(C)** exposure: ebi-a-GCST004131; outcome: ukb-a-88 **(D)** exposure: ebi-a-GCST004131; outcome: finn-b-M13_ANKYLOSPON_STRICT **(E)** exposure: finn-b-K11_IBD_STRICT; outcome: ukb-a-88 **(F)** exposure: finn-b-K11_IBD_STRICT; outcome: finn-b-M13_ANKYLOSPON_STRICT.

The IBD datasets “ieu-a-31”, “ebi-a-GCST004131”, and “finn-b-K11_IBD_STRICT”, along with the AS datasets “ukb-a-88” and “finn-b-M13_ANKYLOSPON_STRICT” were utilized for MR analysis (23–25). The IBD datasets encompassed 308,662 samples, including 41,677 patients, while the AS dataset comprised 555,189 samples, with 1,567 being patients. A total of 6 MR analyses were conducted across 3 exposure datasets and 2 outcome datasets. All analyses underwent scrutiny via the MR-PRESSO test, MR-EGGER test, and Cochran’s Q test, consistently showing no evidence of pleiotropy or heterogeneity. The results of the 6 MR analyses, based on datasets from various consortiums, yielded significant outcomes. While these results indicated variations in ORs stemming from diverse exposures and outcomes, all ORs were greater than 1, supporting the notion of IBD as a risk factor for AS. **Table 1** and **Figure 2** showed the main results of the 6 sets of MR analyses. More details were provided in the **Supplementary Materials**.

**Table 1:** MR analysis between IBD and AS.

Exposure ID	Outcome ID	Method	P value	OR	95%Cl
ieu-a-31	ukb-a-88	IVW	6.62x10^-06^	1.0006	1.0004-1.0009
ieu-a-31	finn-b-M13_ ANKYLOSPON_STRICT	IVW	6.57x10^-06^	1.3446	1.1822-1.5294
ebi-a-GCST004131	ukb-a-88	IVW	1.17x10^-07^	1.0007	1.0005-1.0010
ebi-a-GCST004131	finn-b-M13_ ANKYLOSPON_STRICT	IVW	7.26x10^-08^	1.3854	1.2304-1.5599
finn-b-K11_IBD_ STRICT	ukb-a-88	IVW	0.03	1.0003	1.00003-1.0005
finn-b-K11_IBD_ STRICT	finn-b-M13_ ANKYLOSPON_STRICT	IVW	1.27x10^-13^	1.6753	1.4616-1.9204

### 3.2 MR analysis: key DEGs in IBD associated with the development of AS

**Figure 3:**
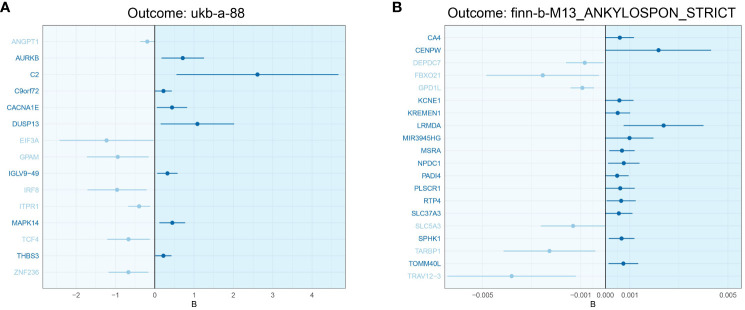
Risk and protective effects of key DGEs with |logFC| > 0.3 **(A)** The key DEGs of outcome ukb-a-88 **(B)** The key DEGs of outcome finn-b-M13_ANKYLOSPON_STRICT.

The analysis revealed 2761 significant DEGs between IBD and healthy controls. In the analysis of primary outcomes, 140 genes were identified to be involved in the development of AS. Of these 140 key genes, 66 exhibited OR values exceeding 1, classifying them as risk genes, while 74 displayed OR values below 1, denoting protective genes. However, not all risk genes were up-regulated in IBD. In fact, only 30 risk genes demonstrated up-regulation, and 44 protective genes exhibited down-regulation within the study. In the analysis of validation outcome, 112 genes were identified as contributors to AS development, with 54 being risk genes and 58 protective genes. Intriguingly, only five risk genes (TINF2, IMMT, ZNF408, NEU1, and APOM) and three protective genes (ANKRD13C, KDELR2, and ZKSCAN8) held significance in both analyses. **Figure 3** showed the risk and protective effects of key DGEs with |logFC| > 0.3. The OR, P-values, LogFC and other information for these genes were provided in the **Supplementary Materials**.

As for the protein levels, due to limited availability of pQTL data sources, verification was only conducted for a subset of the risk genes. In the primary outcome analysis, proteomic data were accessible for 19 out of the 66 risk genes. Among these, APOM and TP53I11 exhibited a positive causal association with AS at the protein level. For the validation outcome analysis, pQTL data were found for 16 out of the 54 risk genes. Among these genes, only DYNLL2 demonstrated positive results on protein levels that aligned with eQTL analysis findings. However, considering the limitations of MR Analysis, the possibility that these proteins are involved in the pathogenesis of AS cannot be completely ruled out even in the absence of positive results. Detailed results of MR Analysis of pQTL were provided in **Supplementary Materials**.

### 3.3 ssGSEA analysis: patients with active IBD showed higher risk scores

Given the occurrence of both up-regulation and down-regulation among risk and protective genes, and considering the limited number of genes that were significant in both outcomes, the study then shifted its focus towards comprehending the overall regulatory patterns of these genes within active and inactive patients. The set of 140 pivotal genes were categorized as 66 risk genes and 74 protective genes based on their respective OR values. Subsequently, individual ssGSEA scores were calculated to examine the enrichment profiles of these two gene categories across distinct phenotypic samples. Likewise the 112 genes assessed in the validation outcome analysis were categorized as 54 risk genes and 58 protective genes, and their corresponding scores were calculated.

**Figure 4:**
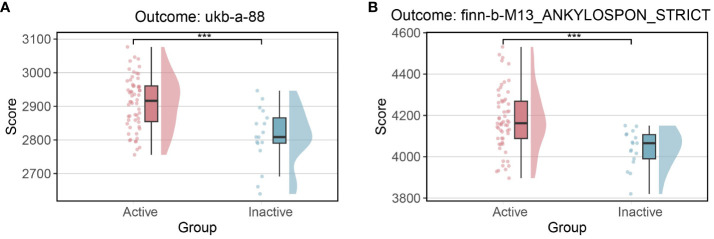
Risk scores between active and inactive patients **(A)** Risk scores in outcome ukb-a-88 **(B)** Risk scores in outcome finn-b-M13_ANKYLOSPON_STRICT.

Notably, across both outcome analyses, active patients consistently exhibited significantly higher risk scores compared to their inactive counterparts (as depicted in **Figure 4**). However, inconsistencies were observed in the protective scores between the two outcome analyses. These findings suggested that alterations in risk genes might hold greater significance than those in protective genes in the process of how IBD leading to AS. Moreover, the results indicated that active patients might face an elevated risk compared to inactive patients, emphasizing the clinical implications of disease activity.

### 3.4 Transcription factor analysis: risk transcription factor activity differed between active and inactive patients

**Figure 5:**
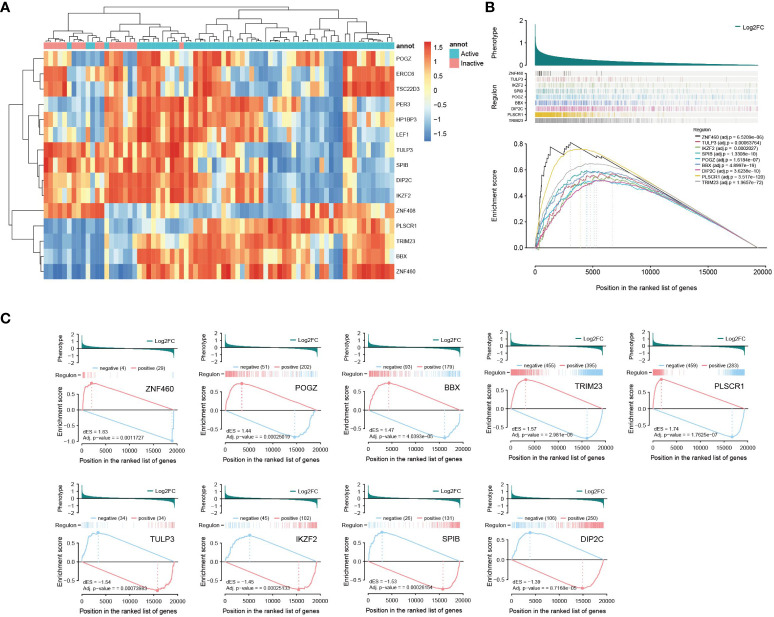
Transcription factor analyses **(A)** Clustering heat map of risk transcription factor activity **(B)** TFs with significant disparities in their regulatory networks between active and inactive patients (one-tailed GSEA) **(C)** Two-tailed GSEA of TFs with significant disparities between active and inactive patients.

Among the 66 risk genes of the primary outcome, 9 TFs were identified, while 7 TFs were identified among the 54 risk genes of the validation outcome, culminating in a total of 15 distinct nonredundant risk TFs. Through one-tailed gene set enrichment analysis of these transcription factors, it became evident that 9 out of the 15 risk TFs exhibited significant disparities in their regulatory networks between active and inactive patients (as illustrated in the **Figure 5B**).

Expanding on this, a two-tailed GSEA was performed on the 9 transcription factors that were identified as significant in the previous analysis. The results revealed a divergence in the effects of these transcription factors. Specifically, genes positively correlated with TRIM23, ZNF460, POGZ, PLSCR1, and BBX were activated, while genes negatively linked to them were suppressed in active patients compared to inactive patients. Conversely, the remaining four genes—TULP3, IKZF2, SPIB and DIP2C—exhibited contrasting changes in expression patterns (as shown in the **Figure 5C**).

Based on the clustering analysis of transcription factor activity, a noticeable pattern emerged in which active and inactive patients were clearly separated, as illustrated in **Figure 5A**. Analyses of risk TFs hinted at potential disparities between active and inactive IBD patients in the progression leading to AS.

### 3.5 GSEA: inflammatory pathways differed between active and inactive patients

**Figure 6:**
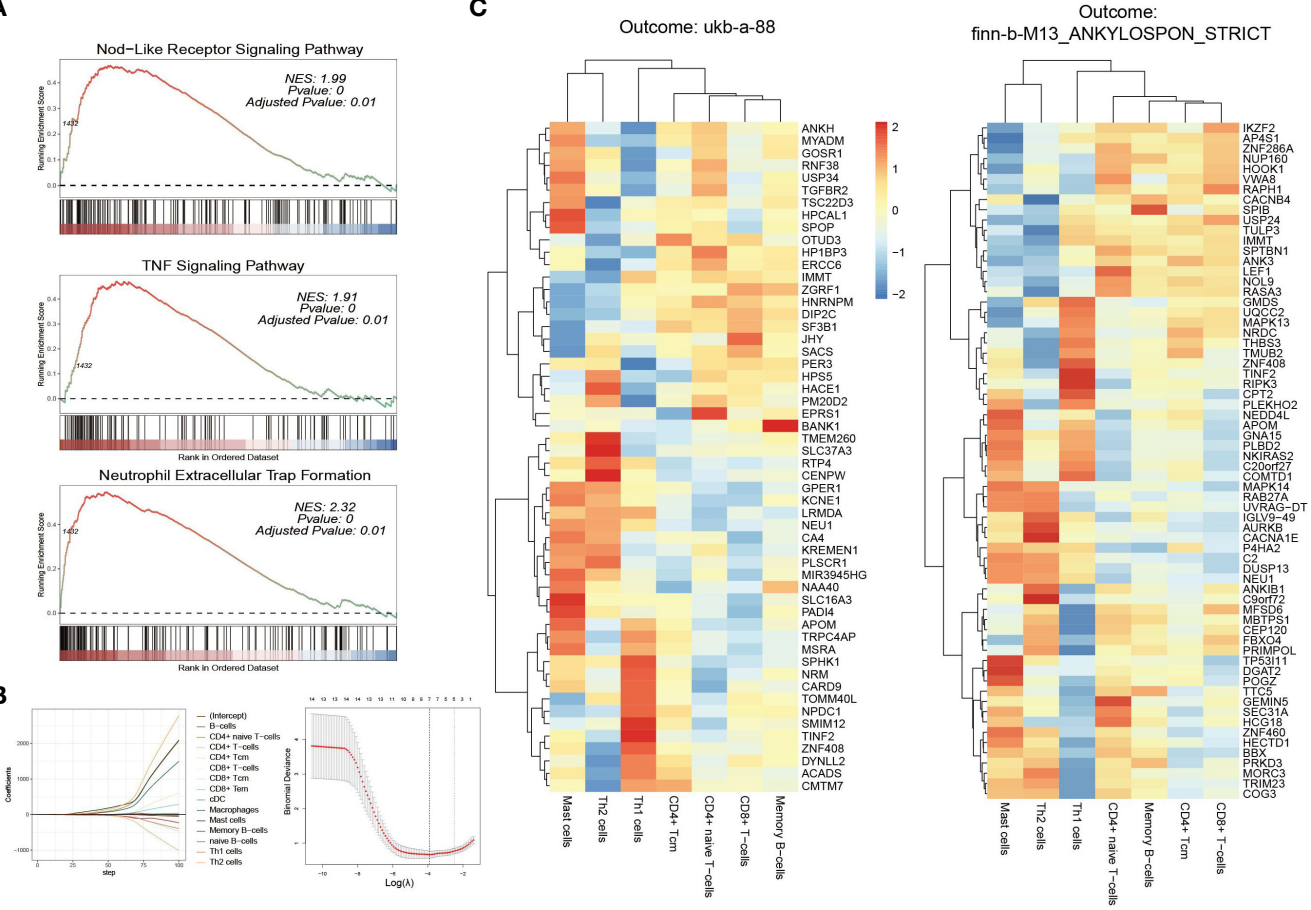
GSEA and Immune cell analyses **(A)** GSEA of “Nod-like receptor signaling pathway,” “neutrophil extracellular trap (NET) formation,” and “TNF signaling pathway,” between active and inactive patients active and inactive patients **(B)** LASSO regression screens key immune cells **(C)** Correlation coefficients between key immune cells and risk genes (left: ukb-a-88; right: finn-b-M13_ANKYLOSPON_STRICT).

According to the results of GSEA analysis, this investigation directed its attention towards three inflammation-associated signaling pathways that demonstrated substantial discrepancies between active and inactive patients. These pathways included the “Nod-like receptor signaling pathway”, “neutrophil extracellular trap (NET) formation,” and “TNF signaling pathway”, all of which exhibited significant activation in active patients compared to their inactive counterparts (as shown in the **Figure 6A**). The combination of these GSEA findings pointed towards a profound interplay between the disease activity of IBD and the heightened activation of inflammatory pathways, potentially exerting an influence on the pathogenesis of AS.

### 3.6 Immune cell enrichment analysis: immune cells closely associated with the disease activity of IBD

Enrichment scores were computed for 34 immune cell types, and subsequently univariate regression analysis was conducted. The results highlighted a total of 14 immune cell types exhibiting significant correlations with disease activity. Out of these 14 cell types, a more comprehensive analysis was performed using LASSO regression (as shown in the **Figure 6B**), resulting in the identification of the 7 most pertinent immune cell types. These included CD4+ naive T-cells, CD4+ Tcm, CD8+ T-cells, Mast cells, Memory B-cells, Th1 cells, and Th2 cells.

Further analysis delved into the association between these seven immune cell types and risk genes, revealing that 63 out of the 66 risk genes exhibited significant correlations with at least one immune cell type. Among these, 39 genes displayed significant associations with CD8+ T-cells, followed by 31 genes with Th1 cells, and 30 genes with CD4+ naive T-cells and CD4+ Tcms each. Memory B-cells were associated with 28 genes, while Mast cells and Th2 cells were linked with 9 and 19 genes, respectively. These findings were consistent in the validation data as well. Out of the 54 risk genes analyzed, 52 were significantly associated with at least one immune cell type. Specifically, CD8+ T-cells were associated with 32 genes, CD4+ naive T-cells with 29 genes, Th1 cells with 27 genes, CD4+ Tcms with 25 genes, memory B-cells with 24 genes, and mast cells with 7 genes. The correlation coefficients between these genes and immune cells were depicted in the **Figure 6C**. Detailed P-values were available in the **Supplementary Materials**.

The analyses of immune cells not only unveiled discrepancies between active and inactive patients but also underscored the close relationship between these immune cell types and risk genes, indicating their potential involvement in the pathogenesis of AS.

## 4 Discussion

This study addressed two pivotal clinical inquiries: does IBD directly lead to the development of AS, and does the activity of IBD exert an influence in this context? Previous studies have not provided sufficient answers due to multiple factors. On one hand, the challenge arose from the inherent difficulty in establishing causal relationships within previous study designs. On the other hand, the intermittent nature of IBD flares and the insidious onset of AS, making it challenging to precisely determine the onset of disease, further complicated the demonstration of the impact of IBD activity on the development of AS.

This study forged a novel approach, the conjunction of Mendelian randomization analysis and transcriptomic data, thereby establishing a linkage between the genetic level and macroscopic phenotypic interactions. This endeavor not only unveiled IBD as an influencing factor in the development of AS but also provided evidence suggesting that active IBD might have an increased potential to precipitate the onset of AS.

Six sets of Mendelian randomization analyses provided relatively robust evidence for IBD being a risk factor for AS. The shared underlying mechanisms of IBD and AS may help elucidate why individuals with IBD are more susceptible to developing AS. Certain genes related to these diseases overlap, with IL23R being a notable example. However, IL23A is associated with IBD but not AS (26, 27). “Gut-synovial axis” hypothesis, implicating environmental and host factors, was an attempt to establish a connection between the intestine and the joint (28). Intestinal dysbiosis and the disruption of epithelial barrier could trigger the activation of innate cells, innate-like cells and specific subsets of T cells, contributing to the pathogenesis of spondyloarthritis (29). Furthermore, immune cell migration initiated in the gut and directed towards other locations was observed in patients with AS. CX3CR1+CD59+ mononuclear phagocytes and type 3 innate lymphoid cells (ILC3s) expanded not only within inflamed gut tissue but also in the peripheral blood, synovial fluid, and bone marrow of patients with AS. The presence of CCR9 in non-intestinal CX3CR1+CD59+ cells and the expression of the α4β7 integrin in ILC3s probably indicate their intestinal origin (30, 31).

The analysis of pathogenic genes presented vastly different landscapes depending on the outcome chosen. Only 8 genes showed consistent results across the analyses of the two outcomes, which could be attributed to the differences in population samples encompassed by the distinct outcomes. Among the five common risk genes, TINF2 is one of six components of the telomere protein complex, also known as shelterin (32). It participates in multiple protein-protein interactions crucial for maintaining the protective function of shelterin at the telomeric ends (33). IMMT constitutes a fundamental element of the mitochondrial contact site complex, which governs the stability of mitochondrial cristae (34). ZNF408 encodes a transcription factor equipped with 10 predicted C2H2-type zinc fingers, believed to be involved in DNA binding (35) and its mutation showed a great impact on retinal vascular development (36, 37). NEU1, a neurophysin, specifically binds to oxytocin and functions as a carrier protein facilitating the transport of hormones from the hypothalamus, where they are synthesized, to the storage site in the pituitary nerve lobe (38). APOM, a prominent member of the apolipoprotein family, serves as a principal carrier of sphingosine-1-phosphate (39). However, APOM’s role extends beyond metabolic and cardiovascular disorders, encompassing contributions to autoimmunity and inflammation (40–42). Among the three shared down-regulated protective genes, ANKRD13C operates as a molecular chaperone for G protein-coupled receptors, overseeing their biogenesis and exit from the endoplasmic reticulum (43). KDELR2, located on the Golgi apparatus, can bind to the K-D-E-L sequence motif and prevent secretion of soluble endoplasmic reticulum-resident proteins (44). ZKSCAN8 encodes a zinc finger protein with KRAB and SCAN domains with little experimental study of its functions. These eight genes exhibit diverse localization and functions, and most importantly, their exploration in IBD and AS remains largely unexplored.

The differences in results of pathogenic genes arising from the selection of distinct outcome data, coupled with the scarcity of research on shared pathogenic genes in IBD and AS, hinted at the need for caution when attributing disease causality to a single gene. Therefore, this study shifted its focus to investigating the broader expression patterns. Through ssGSEA analysis, it was revealed that despite differing sets of risk genes, the overall risk scores for active patients were significantly higher than those for inactive patients. Further analysis of risk transcription factors also supported the distinctions between active and inactive patients.

Subsequently, a question arose: which pathways and immune cell types exhibited significant differences between active and inactive patients? The GSEA analysis presented compelling findings, demonstrating that three crucial inflammatory pathways were significantly activated in active patients, whereas they were not pronounced in inactive patients. The NOD-like receptor (NLR) family of proteins comprises a group of pattern recognition receptors recognized for their role in initiating the initial innate immune response to cellular injury and stress. Several NLRs participate in the formation of inflammasomes (45), with NLRP3 being the most extensively studied among them. NLRP3 and IL-1β were found to be up-regulated in active UC and CD patients compared to those in a quiescent state (46). In addition, the NLRP3 inflammasome was activated in CD patients and in UC patients with long-standing disease (47). However, in animal studies, NLRP3 has demonstrated a bidirectional effect, both promoting inflammation and safeguarding gut integrity. On one hand, NEK7 interacted with NLRP3 to regulate NLRP3 inflammasome activation, thereby modulating the pyroptosis in MODE-K cells and DSS-induced chronic colitis in mice (48). Conversely, Nlrp3(-/-) mice displayed heightened susceptibility to experimental colitis, underscoring its vital role in maintaining intestinal homeostasis (49). There were also treatments that work by inhibiting the NLRP3 inflammasomes (50, 51). Apart from NLRP3, NLRP6 and NLRP1 inflammasomes were also reported to contribute to IBD pathogenesis (52, 53). Meanwhile, an increase of NLRP3 inflammasomes and NLRP3 inflammasome-derived proinflammatory cytokines was observed in peripheral blood mononuclear cells of patients with AS (54). Remarkably, the inflammasome activation in AS was reported to be associated with gut dysbiosis (55), suggesting the potential impact of IBD on AS.

The role of the TNF pathway in both IBD and AS is undeniable, as the remarkable success of anti-TNF therapy serves as compelling evidence that TNF is a pivotal inflammatory factor in both diseases. This also underscores the association between TNF and disease activity in IBD, aligning with the observations of this study.

NETs are intricate extracellular structures, resembling web-like formations, composed of cytosolic and granule proteins assembled on a framework of decondensed chromatin. NETs serve a dual role, offering protection against various pathogens such as bacteria, fungi, viruses, and parasites, while also contributing to the development of immune-related disorders (56). The active involvement of NETs in IBD has become increasingly evident, indicating their capability to exacerbate inflammatory responses and impair intestinal barrier (57–59). Numerous studies substantiated a positive correlation between NET abundance and active disease, with density linked to histopathological severity in IBD cases (58–61). Moreover, several therapeutic approaches exhibited efficacy in ameliorating IBD by targeting NETs (62, 63). As for AS, it was reported that neutrophils from AS patients exhibited heightened formation of NETs containing bioactive IL-17A (64). Elevated IL-17 levels was observed in AS patients, with serum concentrations correlating with disease activity (65, 66). Furthermore, anti-IL-17 therapy has demonstrated significant effectiveness in AS treatment (67). This suggests the possibility that NETs contribute to AS pathogenesis through IL-17, although other potential mechanisms remain inadequately explored.

The study revealed a captivating pattern, with significantly heightened activation of inflammatory pathways in active IBD patients as compared to inactive ones. Considering that AS is also characterized by chronic recurring autoinflammation and is believed to be triggered by infections, this raises a conjecture that the distinct inflammation arising from IBD, separate from infections, may potentially serve as a factor in initiating or exacerbating AS.

The analysis of immune cells revealed that CD8+ T cells and CD4+ T cells (including CD4+ naive T-cells, Th1 cells, and CD4+ Tcms) were likely to be significant contributors, as they exhibited a strong correlation with disease activity. While the role of CD8+ T cells in IBD was controversial, possibly stemming from variations in sources and subsets, it is suggested that activated cytotoxic CD8+ T cells could initiate and contribute to the progression of IBD (68). On the other hand, CD4+ naive T cells can be induced to differentiate into various subpopulations, with Th1 being particularly relevant to this study. Th1 cells release TNF-α and IFN-γ, triggering responses in other immune cells and fostering inflammation. Excessive Th1 responses were observed in IBD patients, with both UC and CD showcasing activated effector Th1 cells (69, 70).

Moreover, CD8+T cells and CD4+T cells are also involved in the pathogenesis of AS. It has been reported that human leukocyte antigen B*27 (HLA-B27), a crucial marker for AS, plays a role in presenting pathogenic peptides to CD8+ T cells (71). Additionally, the autoreactivity of CD8(+) T cells towards HLA-B27-restricted self-epitopes was reported to be associated with AS (72). Comparative data from serum and synovial fluid suggested that cytotoxic phenotype CD8+ T cells are recruited to the joints where they exhibit an activated phenotype (73). A meta-analysis revealed significant increases in the proportions of CD4+ T cells as well as Th1/Th2 ratio in AS patients (74). Furthermore, Th1 cells were found to respond more frequently to conserved E. coli proteins in peripheral blood mononuclear cells and synovial fluid mononuclear cells in AS patients compared to RA patients (75). Additionally, our findings also highlighted a significant association between risk genes and CD8+ T cells as well as CD4+ T cells, thereby suggesting their potential pathogenic role.

In this study, we observed a higher score of AS risk genes in active IBD patients compared to non-active patients, indicating the potential association between disease activity of IBD and the development of AS. Moreover, the clustering analysis revealed that these critical genes primarily contributed to inflammatory pathways, suggesting the potential therapeutic benefits of managing inflammation in individuals with IBD to prevent the onset of AS.

The main constraint of this study lay in the limited data sample source, which introduced potential biases that were challenging to precisely estimate. In addition, the application process of MR analysis also has limitations. The selection of appropriate genetic variation as an IV is crucial to the results of Mendelian randomization analysis, and genetic variants may have horizontal pleiotropy, leading to misjudgments about causation. Additionally, MR Analysis relies on certain assumptions such as strong correlation and independence, which if not met can result in misestimates. To mitigate these potential risks, this study utilized three distinct IBD GWAS datasets as exposure and two separate AS GWAS datasets as outcomes to enhance outcome reliability. IVs that might be associated with confounders or strongly associated with results were also carefully screened out. Furthermore, pleiotropy and heterogeneity were tested by multiple methods. However, despite these efforts, the possibility of errors cannot be entirely dismissed.

In conclusion, this study, through the integration of Mendelian randomization and transcriptome analysis, not only suggested IBD as a potential risk factor for AS but also employed AS-associated risk genes as a bridge, providing novel evidence for the influence of IBD activity on the development of AS.

## Data availability statement

All data are publicly available. Detailed information for these datasets is summarized in **Supplementary Material**.

## Ethics statement

This study was approved by the ethics board of Yueyang Hospital of Integrated Traditional Chinese and Western Medicine. All methods were performed in accordance with the relevant guidelines and regulations.

## Author contributions

YD: Data curation, Formal analysis, Methodology, Writing – original draft. JC: Methodology, Writing – review & editing. RL: Validation, Writing – review & editing. LX: Funding acquisition, Supervision, Validation, Writing – review & editing.

## Supplementary material

Additional File 1
**Table S1** Datasets Information (.doc).

Additional File 2 Detailed information of MR analysis between IBD and AS (.pdf).

Additional File 3 Detailed information of key DEGs (.pdf).

Additional File 4 P-values of the correlation analysis between risk gene and immune cell types (.pdf).

Additional File 5 Detailed information of MR analysis of pQTL (.pdf).
