# Supplementary material for: Inflammatory bowel disease activity threatens ankylosing spondylitis: implications from Mendelian randomization combined with transcriptome analysis
Source: Front Immunol. 2024 Feb 28;15:1289049. doi: 10.3389/fimmu.2024.1289049 (PMC10933069; doi:10.3389/fimmu.2024.1289049)
Supplement: Additional File 1 — Table S1 Datasets Information (.doc). [file DataSheet_1.pdf]

Detailed information of MR analysis between IBD and AS

exposure: ieu-a-31, outcome: ukb-a-88
p1 = 5e-08,r2 = 0.001

before	63snp
remove	reason
rs11209026	directly related to AS
rs4676408	pleiotropy
after	61snp

$`MR-PRESSO results`$`Global Test`$RSSobs
[1] 75.05391

$`MR-PRESSO results`$`Global Test`$Pvalue
[1] 0.1342

Q	Q_df	Q_pval
71.93047	59	0.120313
72.68951	60	0.126017

pleio

0.4332446

results

method	b	se	pval	lo_ci	up_ci	or	or_lci95	or_uci95
MR Egger	0.000301	0.000442	0.499048	-0.00057	0.001167	1.0003	0.9994	1.0012
Weighted median	0.000619	0.000196	0.00162	0.000234	0.001004	1.0006	1.0002	1.0010
Inverse variance weighted	0.000632	0.00014	6.62E-06	0.000357	0.000906	1.0006	1.0004	1.0009
Simple mode	0.000729	0.000451	0.111476	-0.00016	0.001614	1.0007	0.9998	1.0016
Weighted mode	0.000323	0.000345	0.352078	-0.00035	0.000999	1.0003	0.9996	1.0010

exposure: ieu-a-31, outcome: finn-b-M13_ANKYLOSPON_STRICT
p1 = 5e-08,r2 = 0.001

before	62snp
remove	reason
rs11209026	directly related to AS
rs9272514	heterogeneity
after	60snp

$`MR-PRESSO results`$`Global Test`$RSSobs
[1] 76.26574

$`MR-PRESSO results`$`Global Test`$Pvalue
[1] 0.1

Q	Q_df	Q_pval
73.49142	58	0.082594
73.84238	59	0.092363

pleio

0.6006914

results

method	b	se	pval	lo_ci	up_ci	or	or_lci95
MR Egger	0.42412	0.252044	0.097805	-0.06989	0.918127	1.528246	0.9325
Weighted median	0.344123	0.090617	0.000146	0.166514	0.521733	1.410753	1.18118
Inverse variance weighted	0.296115	0.065697	6.57E-06	0.167349	0.424881	1.344624	1.182166
Simple mode	0.41582	0.196866	0.038912	0.029963	0.801677	1.515613	1.030416
Weighted mode	0.429861	0.174231	0.016539	0.088368	0.771353	1.537043	1.09239

exposure: ebi-a-GCST004131, outcome: ukb-a-88
p1 = 5e-08,r2 = 0.001

before	115snp
remove	reason
rs145568234	directly related to AS
rs4676408	pleiotropy
rs11581607	pleiotropy
after	112snp

$`MR-PRESSO results`$`Global Test`$RSSobs
[1] 133.2803

$`MR-PRESSO results`$`Global Test`$Pvalue
[1] 0.101

Q	Q_df	Q_pval
113.9373	98	0.129457
114.5153	99	0.136411

pleio
0.4824066

results

method	b	se	pval	lo_ci	up_ci	or	or_lci95	or_uci95
MR Egger	0.00044	0.00045	0.330412	-0.00044	0.001323	1.000441	0.999558	1.001324
Weighted median	0.000818	0.000187	1.27E-05	0.000451	0.001185	1.000818	1.000451	1.001186
Inverse variance weighted	0.000742	0.00014	1.17E-07	0.000468	0.001017	1.000742	1.000468	1.001017
Simple mode	0.001131	0.000425	0.009045	0.000299	0.001964	1.001132	1.000299	1.001966
Weighted mode	0.000957	0.000376	0.012574	0.000219	0.001694	1.000957	1.000219	1.001696

exposure: ebi-a-GCST004131, outcome: finn-b-M13_ANKYLOSPON_STRICT
p1 = 5e-08,r2 = 0.001

before	114snp
remove	reason
rs145568234	directly related to AS
rs1864239	pleiotropy
rs140933577	pleiotropy
rs143210366	pleiotropy
rs11768365	pleiotropy
after	109snp

$`MR-PRESSO results`$`Global Test`$RSSobs
[1] 126.2648

$`MR-PRESSO results`$`Global Test`$Pvalue
[1] 0.1492

Q	Q_df	Q_pval
108.2363	95	0.166816
108.3662	96	0.182887

pleio
0.7363617

results

method	b	se	pval	lo_ci	up_ci	or	or_lci95	or_uci95
MR Egger	0.27377	0.166168	0.102748	-0.05192	0.599459	1.314912	0.949406	1.821133
Weighted median	0.331593	0.092021	0.000314	0.151231	0.511955	1.393186	1.163266	1.668549
Inverse variance weighted	0.325985	0.06054	7.26E-08	0.207326	0.444644	1.385395	1.230384	1.559934
Simple mode	0.204082	0.206761	0.326102	-0.20117	0.609333	1.226399	0.817774	1.839204
Weighted mode	0.309803	0.14288	0.03261	0.029758	0.589849	1.363157	1.030205	1.803716

exposure: finn-b-K11_IBD_STRICT, outcome: ukb-a-88
p1 = 5e-06,r2 = 0.01

before	64snp
remove	reason
rs3823377	pval.outcome<5e-06
rs145568234	pval.outcome<5e-06
rs2836878	directly related to AS
rs1391371	pleiotropy
rs12132349	pleiotropy
rs2964085	pleiotropy
rs56016561	pleiotropy
after	58snp

$`MR-PRESSO results`$`Global Test`$RSSobs
[1] 59.42872

$`MR-PRESSO results`$`Global Test`$Pvalue
[1] 0.4298

Q	Q_df	Q_pval
51.51359	53	0.532193
51.87916	54	0.556616

pleio
0.5480107

results

method	b	se	pval	lo_ci	up_ci	or	or_lci95	or_uci95
MR Egger	0.000105	0.000327	0.750051	-0.00054	0.000745	1.000105	0.999465	1.000745
Weighted median	0.000184	0.000207	0.373931	-0.00022	0.000591	1.000184	0.999778	1.000591
Inverse variance weighted	0.000286	0.000131	0.029026	2.92E-05	0.000542	1.000286	1.000029	1.000542
Simple mode	0.000212	0.00043	0.624833	-0.00063	0.001055	1.000212	0.999368	1.001056
Weighted mode	0.000105	0.000311	0.735624	-0.0005	0.000715	1.000106	0.999496	1.000715

exposure: finn-b-K11_IBD_STRICT, outcome: finn-b-M13_ANKYLOSPON_STRICT
p1 = 5e-06,r2 = 0.01

before	67snp
remove	reason
rs35978646	pval.outcome<5e-06
rs2077163	pval.outcome<5e-06
rs181316459	pval.outcome<5e-06
rs62443225	pval.outcome<5e-06
rs3823377	pval.outcome<5e-06
rs2836878	directly related to AS
rs116544448	pleiotropy
rs1391371	pleiotropy
rs57882523	pleiotropy
rs6966158	pleiotropy
rs7781433	pleiotropy
rs10239619	pleiotropy
rs9617090	pleiotropy
rs145568234	pleiotropy
rs7936070	pleiotropy
rs10807943	pleiotropy
rs116929608	pleiotropy
after	50snp

$`MR-PRESSO results`$`Global Test`$RSSobs
[1] 66.69811

$`MR-PRESSO results`$`Global Test`$Pvalue
[1] 0.1122

Q	Q_df	Q_pval
52.89113	46	0.225375
57.49337	47	0.140406

pleio
0.05135176

results

method	b	se	pval	lo_ci	up_ci	or	or_lci95	or_uci95
MR Egger	0.913729	0.209945	7.44E-05	0.502236	1.325222	2.493604	1.652412	3.76302
Weighted median	0.524009	0.098245	9.62E-08	0.331449	0.716569	1.688784	1.392985	2.047396
Inverse variance weighted	0.516017	0.069645	1.27E-13	0.379512	0.652522	1.675342	1.461571	1.920378
Simple mode	0.432783	0.245978	0.085012	-0.04933	0.9149	1.541541	0.951863	2.496526
Weighted mode	0.445112	0.237855	0.067527	-0.02108	0.911309	1.560666	0.979137	2.487576
