# Supplementary material for: Inflammatory bowel disease activity threatens ankylosing spondylitis: implications from Mendelian randomization combined with transcriptome analysis
Source: Front Immunol. 2024 Feb 28;15:1289049. doi: 10.3389/fimmu.2024.1289049 (PMC10933069; doi:10.3389/fimmu.2024.1289049)
Supplement: Additional File 2 — Detailed information of MR analysis between IBD and AS (.pdf). [file DataSheet_2.pdf]

Detailed information of key DEGs

Key DEGs for finn-b-M13_ ANKYLOSPON_STRICT

id.exposure	nsnp	b	se	pval	SYMBOL	logFC	or	or_lci95	or_uci95
ENSG00000001629	2	0.28119	0.13576	0.03834	ANKIB1	-0.2506	1.3247	1.01522	1.72853
ENSG00000004487	rs67056484	-1.1687	0.4886	0.01676	KDM1A	-0.2446	0.31076	0.11927	0.80973
ENSG00000030066	rs6485784	0.61256	0.30128	0.04203	NUP160	-0.2086	1.84516	1.02231	3.33031
ENSG00000030419	rs34053836	1.01226	0.49381	0.04037	IKZF2	-0.2471	2.75182	1.04539	7.24372
ENSG00000040633	3	-0.435	0.20759	0.03613	PHF23	0.27036	0.64726	0.4309	0.97227
ENSG00000042493	4	-0.2312	0.10093	0.02197	CAPG	0.31351	0.79357	0.65115	0.96716
ENSG00000049759	2	0.38498	0.19607	0.04959	NEDD4L	-0.1231	1.46958	1.00068	2.1582
ENSG00000058056	rs2276803	-0.5241	0.20273	0.00973	USP13	-0.2241	0.59206	0.39792	0.88091
ENSG00000060558	3	0.62685	0.28765	0.02932	GNA15	0.28493	1.87171	1.06508	3.28921
ENSG00000062282	4	0.77667	0.30145	0.00998	DGAT2	0.26109	2.17421	1.20421	3.92557
ENSG00000069974	2	0.64417	0.30419	0.03421	RAB27A	0.20091	1.90441	1.04912	3.45697
ENSG00000072682	rs11955347	1.08556	0.43961	0.01354	P4HA2	0.15887	2.9611	1.25097	7.00906
ENSG00000074603	rs12594547	-0.5829	0.25177	0.02059	DPP8	-0.14	0.55825	0.34082	0.91441
ENSG00000078246	rs76487928	1.33988	0.5637	0.01746	TULP3	-0.1995	3.81857	1.26494	11.5274
ENSG00000078618	3	0.31043	0.13216	0.01883	NRDC	0.11277	1.36402	1.05274	1.76734
ENSG00000079393	2	1.08527	0.47608	0.02263	DUSP13	0.51275	2.96025	1.16434	7.5262
ENSG00000082516	rs1390600	1.15263	0.54564	0.03465	GEMIN5	-0.3149	3.1665	1.08672	9.22658
ENSG00000083642	3	-0.839	0.35151	0.01699	PDS5B	-0.2047	0.43212	0.21697	0.86062
ENSG00000083844	2	-0.6906	0.25665	0.00713	ZNF264	-0.2334	0.50127	0.30312	0.82896
ENSG00000092148	rs112174347	1.08744	0.41941	0.00952	HECTD1	-0.2134	2.96667	1.30396	6.74955
ENSG00000092330	rs3129720	2.71011	0.43259	3.73E-10	TINF2	0.21069	15.031	6.43817	35.0923
ENSG00000097021	2	-0.4069	0.20477	0.04693	ACOT7	0.20015	0.66573	0.44565	0.9945
ENSG00000097033	6	-0.3385	0.17242	0.04965	SH3GLB1	0.18616	0.71287	0.50845	0.99948
ENSG00000099783	rs9268895	-2.1552	0.65353	0.00097	HNRNPM	-0.2187	0.11588	0.03219	0.41716
ENSG00000100478	rs7149411	1.13495	0.5182	0.02851	AP4S1	-0.0962	3.11103	1.12669	8.59025
ENSG00000101220	5	0.23241	0.09307	0.01252	C20orf27	0.23091	1.26164	1.05126	1.51412
ENSG00000102763	2	0.73996	0.29744	0.01285	VWA8	-0.1604	2.09586	1.16997	3.75446
ENSG00000105518	6	-0.2752	0.13716	0.04481	TMEM20	0.15344	0.75942	0.5804	0.99365
ENSG00000107581	rs10886384	-1.2257	0.61021	0.04458	EIF3A	-0.3015	0.29356	0.08877	0.97077
ENSG00000109944	rs17126930	-0.2969	0.15023	0.04815	JHY	-0.2297	0.74314	0.55359	0.99759
ENSG00000112062	3	0.447	0.16746	0.0076	MAPK14	0.43322	1.56362	1.12613	2.17107
ENSG00000112685	4	-0.2348	0.09302	0.01158	EXOC2	-0.2124	0.7907	0.65891	0.94884
ENSG00000112699	4	0.52861	0.21892	0.01575	GMDS	0.15641	1.69657	1.10465	2.60566
ENSG00000113595	2	0.69994	0.28256	0.01324	TRIM23	-0.2687	2.01364	1.15734	3.5035
ENSG00000114439	rs1697689	0.4685	0.20522	0.02243	BBX	-0.2242	1.59759	1.06852	2.38863
ENSG00000115306	5	0.29364	0.12717	0.02094	SPTBN1	-0.1884	1.3413	1.04539	1.72098
ENSG00000115825	4	0.25662	0.12245	0.03611	PRKD3	-0.2951	1.29256	1.01676	1.64318
ENSG00000118454	rs3795695	-0.9714	0.37792	0.01016	ANKRD1	-0.2314	0.37856	0.18049	0.79401
ENSG00000119927	rs79259123	-0.9395	0.40015	0.01888	GPAM	-0.3557	0.39082	0.17838	0.85622
ENSG00000120093	2	-0.3019	0.13416	0.0244	HOXB3	-0.167	0.73938	0.56842	0.96175
ENSG00000124532	2	-0.3489	0.1406	0.01309	MRS2	-0.1489	0.70547	0.53555	0.92932
ENSG00000126804	rs13329053	-1.4923	0.73233	0.04157	ZBTB1	-0.2952	0.22484	0.05352	0.94462
ENSG00000129465	2	0.7056	0.29227	0.01577	RIPK3	0.2801	2.02507	1.14197	3.59108
ENSG00000129467	rs2243891	-1.3599	0.61754	0.02765	ADCY4	0.40372	0.25668	0.07651	0.86109
ENSG00000130856	rs62113105	-0.6691	0.25845	0.00963	ZNF236	-0.3164	0.51217	0.30862	0.84998
ENSG00000132305	4	0.33028	0.123	0.00725	IMMT	-0.1796	1.39136	1.09331	1.77067
ENSG00000133313	4	-0.1906	0.09545	0.04588	CNDP2	0.16342	0.8265	0.68549	0.99653
ENSG00000134056	rs6450023	-1.527	0.74392	0.0401	MRPS36	0.19932	0.21718	0.05053	0.93336
ENSG00000134709	2	0.52258	0.16095	0.00117	HOOK1	-0.2763	1.68637	1.23012	2.31185
ENSG00000134987	2	-1.1609	0.55668	0.03703	WDR36	-0.2201	0.31319	0.10519	0.93253
ENSG00000135636	3	-0.401	0.15075	0.00781	DYSF	0.32136	0.66964	0.49833	0.89983
ENSG00000136152	rs2985952	0.60678	0.27154	0.02544	COG3	-0.2083	1.83452	1.07742	3.12364

ENSG00000136240	5	-0.1532	0.06572	0.01973	KDELR2	-0.1718	0.85794	0.75425	0.97589
ENSG00000136250	6	-0.1535	0.07179	0.03251	AOAH	0.16219	0.85771	0.74513	0.9873
ENSG00000136319	2	0.39037	0.18705	0.03689	TTC5	-0.3023	1.47753	1.02404	2.13182
ENSG00000136848	2	-1.1705	0.50957	0.02162	DAB2IP	-0.1395	0.31022	0.11426	0.84221
ENSG00000137288	rs2296330	1.54699	0.31319	7.83E-07	UQCC2	0.27359	4.69733	2.54249	8.67845
ENSG00000138190	rs7909581	-1.3219	0.48754	0.0067	EXOC6	0.26735	0.26662	0.10254	0.69326
ENSG00000138674	rs28539166	1.69565	0.8473	0.04537	SEC31A	-0.2542	5.4502	1.03555	28.6849
ENSG00000138795	7	1.18775	0.32857	0.0003	LEF1	-0.259	3.27968	1.72246	6.24471
ENSG00000138835	rs7854524	-0.4407	0.17005	0.00955	RGS3	0.27323	0.64358	0.46116	0.89817
ENSG00000140943	3	0.25707	0.12293	0.03651	MBTPS1	-0.1586	1.29314	1.01625	1.64546
ENSG00000140968	7	-0.9578	0.3841	0.01265	IRF8	-0.4081	0.38374	0.18075	0.81469
ENSG00000143382	3	-0.2778	0.12699	0.02868	ADAMTS	0.55683	0.75742	0.59052	0.97148
ENSG00000143442	2	0.46463	0.19082	0.0149	POGZ	-0.2748	1.59142	1.09485	2.31322
ENSG00000145703	8	-0.2157	0.10087	0.03245	IQGAP2	-0.2029	0.80595	0.66138	0.98213
ENSG00000146409	5	-0.1457	0.06165	0.01811	SLC18B1	-0.2648	0.86442	0.76604	0.97544
ENSG00000147894	7	0.22164	0.10757	0.03936	C9orf72	0.31883	1.24812	1.01086	1.54106
ENSG00000148335	2	-0.752	0.34096	0.02742	NTMT1	0.1844	0.47142	0.24164	0.91969
ENSG00000148655	4	-0.9136	0.39274	0.02	LRMDA	0.4481	0.40107	0.18574	0.86601
ENSG00000150995	4	-0.3971	0.14197	0.00515	ITPR1	-0.3087	0.67224	0.50896	0.88791
ENSG00000151150	4	0.19988	0.0993	0.04412	ANK3	-0.2965	1.22126	1.00528	1.48364
ENSG00000151176	2	0.57456	0.26881	0.03256	PLBD2	0.25149	1.77635	1.04885	3.00844
ENSG00000151576	rs9823071	-1.3266	0.63941	0.03801	QTRT2	-0.2339	0.26538	0.07579	0.92925
ENSG00000151690	6	0.15213	0.0755	0.0439	MFSD6	-0.2741	1.16432	1.00417	1.35
ENSG00000151876	rs604472	1.23581	0.55	0.02464	FBXO4	-0.2532	3.44115	1.17095	10.1128
ENSG00000154188	6	-0.1896	0.08987	0.03492	ANGPT1	-0.3242	0.82731	0.69369	0.98667
ENSG00000154813	3	-0.5075	0.18403	0.00582	DPH3	0.21256	0.60203	0.41973	0.8635
ENSG00000154945	rs9889919	-0.7264	0.35754	0.04219	ANKRD4	-0.2877	0.48365	0.23998	0.97471
ENSG00000155974	rs11176169	-1.1188	0.45091	0.0131	GRIP1	-0.2015	0.32668	0.13499	0.79057
ENSG00000156711	2	1.08042	0.39128	0.00576	MAPK13	0.25708	2.94591	1.36822	6.34281
ENSG00000157184	2	0.5055	0.21536	0.01892	CPT2	0.15621	1.65781	1.08696	2.52846
ENSG00000159256	2	0.31882	0.12104	0.00844	MORC3	-0.2322	1.37551	1.08501	1.74378
ENSG00000160679	2	-0.2937	0.13508	0.02971	CHTOP	-0.1032	0.74553	0.57211	0.97151
ENSG00000160685	rs905938	-0.969	0.41052	0.01825	ZBTB7B	0.19995	0.37947	0.16972	0.84842
ENSG00000162402	2	0.82431	0.24713	0.00085	USP24	-0.1972	2.28032	1.40486	3.70133
ENSG00000162408	rs11122089	0.41781	0.19084	0.02858	NOL9	-0.1844	1.51863	1.04473	2.2075
ENSG00000162434	rs310233	-1.7058	0.67484	0.01148	JAK1	-0.2801	0.18163	0.04839	0.68176
ENSG00000163877	4	-0.3238	0.13552	0.01689	SNIP1	-0.2059	0.72342	0.55467	0.94352
ENSG00000164306	2	0.70863	0.31689	0.02534	PRIMPOL	-0.3954	2.03121	1.09146	3.78009
ENSG00000165644	2	0.3605	0.14506	0.01295	COMTD1	0.24189	1.43404	1.07915	1.90565
ENSG00000165995	rs72787951	-0.5621	0.28595	0.04933	CACNB2	-0.2187	0.57001	0.32544	0.99837
ENSG00000166278	rs114502302	2.61248	1.05063	0.0129	C2	0.39316	13.6328	1.73887	106.881
ENSG00000166557	3	-0.5091	0.21448	0.0176	TMED3	0.28481	0.60101	0.39474	0.91507
ENSG00000168256	2	0.3718	0.18591	0.04551	NKIRAS2	0.18453	1.45034	1.00745	2.08793
ENSG00000168566	4	-0.3162	0.12787	0.01341	SNRNP48	-0.1802	0.72893	0.56734	0.93654
ENSG00000168591	rs11079983	0.74547	0.36318	0.04011	TMUB2	0.13267	2.10743	1.03421	4.29433
ENSG00000168944	2	0.73751	0.28122	0.00873	CEP120	-0.2355	2.09073	1.20481	3.62807
ENSG00000169231	4	0.22022	0.10463	0.0353	THBS3	0.37695	1.24636	1.01527	1.53003
ENSG00000170027	4	-0.5063	0.12769	7.34E-05	YWHAG	-0.1907	0.60274	0.46929	0.77414
ENSG00000170846	4	-0.4841	0.2348	0.03922	LOC9362	-0.299	0.61623	0.38893	0.97637
ENSG00000173166	2	0.91457	0.4426	0.03879	RAPH1	-0.1981	2.49571	1.0482	5.94212
ENSG00000173209	4	-0.2324	0.11328	0.04023	AHSA2P	-0.154	0.79264	0.63482	0.98971
ENSG00000173473	rs78216580	-1.3147	0.55271	0.01737	SMARCC	-0.2292	0.26854	0.0909	0.79339
ENSG00000173928	rs651774	-0.5833	0.28607	0.04144	SWSAP1	0.20125	0.55803	0.31853	0.97762
ENSG00000174791	4	-0.302	0.12028	0.01203	RIN1	0.34113	0.73931	0.58405	0.93586
ENSG00000175213	rs9264942	18.1048	0.92267	1.00E-85	ZNF408	0.23314	7.3E+07	1.2E+07	4.4E+08

ENSG00000175274	3	0.4625	0.22647	0.04113	TP53I11	0.29505	1.58804	1.01879	2.47537
ENSG00000176473	5	-0.2126	0.09524	0.02558	WDR25	0.25212	0.80846	0.67079	0.97438
ENSG00000178999	rs12603646	0.71231	0.2756	0.00975	AURKB	0.36472	2.03869	1.18784	3.49899
ENSG00000182197	2	-0.3404	0.13932	0.01454	EXT1	0.41516	0.71145	0.54144	0.93485
ENSG00000182389	8	0.39067	0.1888	0.03852	CACNB4	-0.2124	1.47797	1.02084	2.13981
ENSG00000183161	rs408199	-0.631	0.24476	0.00994	FANCF	-0.1539	0.53207	0.32933	0.85962
ENSG00000183401	rs651774	-0.3863	0.18945	0.04144	CCDC159	0.18683	0.67956	0.46878	0.98512
ENSG00000185621	rs9832681	-0.6395	0.27516	0.02011	LMLN	-0.1535	0.52754	0.30763	0.90464
ENSG00000185989	3	0.89856	0.37252	0.01586	RASA3	-0.1662	2.45607	1.18344	5.09724
ENSG00000187607	rs9890181	0.26325	0.13218	0.04642	ZNF286A	-0.1398	1.30115	1.00418	1.68595
ENSG00000189114	rs79016999	-0.6915	0.31013	0.02577	BLOC1S3	-0.1585	0.50084	0.27272	0.91977
ENSG00000196628	9	-0.6669	0.27554	0.01551	TCF4	-0.5081	0.51331	0.29912	0.8809
ENSG00000197019	rs75569110	-0.3478	0.14662	0.01767	SERTAD1	0.22995	0.70621	0.52982	0.94133
ENSG00000197208	7	-0.315	0.12411	0.01114	SLC22A4	0.46941	0.72979	0.57221	0.93076
ENSG00000197714	rs12463076	0.5167	0.23461	0.02764	ZNF460	-0.1863	1.67649	1.05851	2.65527
ENSG00000198042	rs8685	-0.6239	0.21676	0.004	MAK16	-0.2263	0.53584	0.35037	0.81949
ENSG00000198216	4	0.43958	0.19615	0.02502	CACNA1E	0.40378	1.55205	1.05668	2.27965
ENSG00000198265	2	-0.5478	0.21098	0.00943	HELZ	-0.2915	0.57825	0.38241	0.87439
ENSG00000198315	rs9366718	-4.0612	1.03862	9.22E-05	ZKSCAN3	-0.1926	0.01723	0.00225	0.13193
ENSG00000198824	rs7330717	-1.6035	0.77299	0.03804	CHAMP1	-0.2393	0.2012	0.04422	0.91538
ENSG00000204264	2	-0.4168	0.17005	0.01424	PSMB8	0.16978	0.65913	0.4723	0.91986
ENSG00000204386	rs3130490	2.51461	0.65155	0.00011	NEU1	0.25124	12.3618	3.44726	44.3293
ENSG00000204444	rs3115663	1.75315	0.49647	0.00041	APOM	0.29798	5.77276	2.18163	15.2751
ENSG00000213588	rs456993	-1.8779	0.38108	8.31E-07	ZBTB9	-0.1488	0.15291	0.07245	0.3227
ENSG00000213722	2	-0.9082	0.45692	0.04686	DDAH2	0.50698	0.40326	0.16468	0.98748
ENSG00000223350	2	0.32335	0.13131	0.01379	IGLV9-49	0.5906	1.38175	1.06822	1.78731
ENSG00000229833	rs794081	-0.3472	0.17375	0.04566	PET100	0.35402	0.70663	0.50268	0.99332
ENSG00000231074	3	1.61687	0.78007	0.0382	HCG18	-0.2846	5.03728	1.09192	23.2382
ENSG00000241839	4	0.21561	0.10661	0.04313	PLEKHO2	0.20181	1.24062	1.00667	1.52894
ENSG00000251230	3	-0.5706	0.22525	0.0113	MIR3945I	0.67796	0.56517	0.36345	0.87886
ENSG00000255507	rs67233017	1.64866	0.68606	0.01626	UVRAG-I	0.28921	5.2	1.35523	19.9523
ENSG00000269404	6	0.2755	0.12338	0.02555	SPIB	-0.4831	1.3172	1.03425	1.67755
ENSG00000184281	3	-2.0374	0.781	0.00909	TSSC4	0.25058	0.13036	0.02821	0.6025

Key DEGs for ukb-a-88

id.exposure	nsnp	b	se	pval	SYMBOL	logFC	or	or_lci95	or_uci95
ENSG00000025770	rs470120	-0.0032	0.00144	0.02671	NCAPH2	0.21861	0.99683	0.99403	0.99963
ENSG00000049246	6	0.00058	0.00026	0.02621	PER3	-0.242	1.00058	1.00007	1.00109
ENSG00000051108	3	-0.0012	0.00035	0.00067	HERPUD	-0.1457	0.99882	0.99814	0.9995
ENSG00000058272	rs1919585	-0.0039	0.00144	0.00634	PPP1R12/	-0.1779	0.99609	0.99328	0.9989
ENSG00000121690	rs4756561	-0.0008	0.00039	0.03158	DEPDC7	-0.3257	0.99916	0.9984	0.99993
ENSG00000065883	2	-0.0015	0.00046	0.00156	CDK13	-0.2195	0.99854	0.99764	0.99945
ENSG00000070269	rs12589117	0.00171	0.00078	0.02821	TMEM26	0.23942	1.00172	1.00018	1.00325
ENSG00000076321	2	-0.0013	0.00056	0.0253	KLHL20	-0.1615	0.99875	0.99765	0.99984
ENSG00000080503	2	-0.0021	0.00079	0.00873	SMARCA	-0.2905	0.99794	0.9964	0.99948
ENSG00000085382	rs6922441	0.00195	0.00091	0.031	HACE1	-0.4045	1.00195	1.00018	1.00373
ENSG00000087077	2	-0.0012	0.00045	0.00633	TRIP6	0.35848	0.99878	0.99791	0.99966
ENSG00000088882	2	-0.0026	0.00092	0.00541	CPXM1	0.19584	0.99744	0.99564	0.99924
ENSG00000089351	6	-0.0007	0.00025	0.00505	GRAMD1	0.27766	0.99931	0.99882	0.99979
ENSG00000092330	rs3129720	0.00454	0.00092	8.53E-07	TINF2	0.21069	1.00455	1.00273	1.00636
ENSG00000096063	8	-0.0006	0.00024	0.00641	SRPK1	0.41602	0.99936	0.99889	0.99982
ENSG00000099783	rs9268895	0.00581	0.00144	5.32E-05	HNRNPM	-0.2187	1.00582	1.003	1.00866
ENSG00000100320	rs7289456	-0.0043	0.00144	0.00288	RBFOX2	-0.1015	0.99572	0.99291	0.99853
ENSG00000100991	6	0.00044	0.00022	0.04473	TRPC4AF	0.1618	1.00044	1.00001	1.00086
ENSG00000105379	3	-0.001	0.00037	0.00746	ETFB	0.14211	0.99902	0.9983	0.99974
ENSG00000107281	3	0.00075	0.00033	0.02072	NPDC1	0.35725	1.00075	1.00012	1.00139
ENSG00000108587	2	0.00192	0.00096	0.04599	GOSR1	-0.213	1.00193	1.00003	1.00382
ENSG00000109944	rs17126930	0.00063	0.00031	0.04149	JHY	-0.2297	1.00063	1.00002	1.00123
ENSG00000110583	rs4980499	0.00219	0.00079	0.0058	NAA40	-0.2514	1.00219	1.00063	1.00376
ENSG00000110756	2	0.00066	0.00031	0.03427	HPS5	-0.1658	1.00066	1.00005	1.00126
ENSG00000112053	9	-0.0006	0.00026	0.03565	SLC26A8	1.1379	0.99945	0.99893	0.99996
ENSG00000113971	4	-0.0004	0.00017	0.00911	NPHP3	-0.2655	0.99956	0.99923	0.99989
ENSG00000114054	4	-0.0006	0.00019	0.00269	PCCB	0.24379	0.99942	0.99904	0.9998
ENSG00000115459	rs10460585	-0.0019	0.00095	0.0481	ELMOD3	0.17607	0.99812	0.99627	0.99998
ENSG00000115464	rs10496091	0.00172	0.00062	0.00559	USP34	-0.1736	1.00172	1.0005	1.00294
ENSG00000115524	rs3980095	0.00174	0.00071	0.01455	SF3B1	-0.158	1.00175	1.00035	1.00315
ENSG00000115756	4	0.00055	0.00025	0.0283	HPCAL1	0.1917	1.00055	1.00006	1.00104
ENSG00000117713	2	-0.0008	0.00026	0.00137	ARID1A	-0.185	0.99918	0.99868	0.99968
ENSG00000117984	4	-0.0014	0.00067	0.03341	CTSD	0.32999	0.99858	0.99727	0.99989
ENSG00000118260	3	-0.0012	0.00059	0.03978	CREB1	-0.2039	0.99879	0.99764	0.99994
ENSG00000118454	rs3795695	-0.0013	0.0005	0.00793	ANKRD1	-0.2314	0.99868	0.9977	0.99965
ENSG00000121067	rs10514970	0.00462	0.00193	0.01657	SPOP	-0.1464	1.00463	1.00084	1.00843
ENSG00000135108	rs4766834	-0.0025	0.00117	0.0294	FBXO21	-0.4426	0.99746	0.99517	0.99975
ENSG00000122971	rs56178989	0.00359	0.00178	0.04343	ACADS	0.27705	1.00359	1.00011	1.00709
ENSG00000127483	2	0.00184	0.00084	0.02932	HP1BP3	-0.2347	1.00184	1.00018	1.0035
ENSG00000128512	5	-0.0015	0.00071	0.03545	DOCK4	0.55404	0.9985	0.99711	0.9999
ENSG00000132305	4	0.00056	0.00022	0.01064	IMMT	-0.1796	1.00056	1.00013	1.00099
ENSG00000133328	rs11231521	-0.0016	0.00076	0.03575	PLAAT2	0.25608	0.99841	0.99692	0.99989
ENSG00000133706	2	-0.0011	0.00047	0.01622	LARS1	-0.2059	0.99888	0.99797	0.99979
ENSG00000152642	4	-0.0009	0.00024	0.00012	GPD1L	-0.3331	0.99907	0.99859	0.99954
ENSG00000135269	3	-0.0007	0.00025	0.00608	TES	-0.2198	0.99932	0.99883	0.99981
ENSG00000135828	3	-0.0007	0.00033	0.03561	RNASEL	0.15974	0.99931	0.99867	0.99995
ENSG00000136240	5	-0.0003	0.00017	0.03906	KDELR2	-0.1718	0.99966	0.99933	0.99998
ENSG00000136514	2	0.00065	0.0003	0.03263	RTP4	0.53596	1.00065	1.00005	1.00124
ENSG00000136628	rs35501509	0.00099	0.00039	0.01098	EPRS1	-0.1877	1.00099	1.00023	1.00175
ENSG00000137075	2	0.00152	0.00064	0.01671	RNF38	-0.1626	1.00153	1.00028	1.00278
ENSG00000137161	rs4714634	-0.0014	0.00067	0.03972	CNPY3	0.34127	0.99862	0.9973	0.99993
ENSG00000137404	3	0.00252	0.00056	6.93E-06	NRM	0.22671	1.00252	1.00142	1.00362
ENSG00000138658	3	0.00071	0.00028	0.01158	ZGRF1	-0.2393	1.00071	1.00016	1.00126

ENSG00000140365	3	-0.0011	0.00042	0.01169	COMMD4	0.16532	0.99894	0.99812	0.99976
ENSG00000140995	4	-0.0014	0.00068	0.04028	DEF8	0.2123	0.9986	0.99726	0.99994
ENSG00000141526	3	0.00065	0.00028	0.02105	SLC16A3	0.24256	1.00065	1.0001	1.00121
ENSG00000146281	2	0.00208	0.00087	0.01745	PM20D2	-0.3158	1.00208	1.00036	1.0038
ENSG00000147894	7	-0.0005	0.00021	0.03188	C9orf72	0.31883	0.99955	0.99914	0.99996
ENSG00000148655	4	0.00238	0.00083	0.00415	LRMDA	0.4481	1.00238	1.00075	1.00401
ENSG00000148943	rs11030027	-0.0014	0.00067	0.03459	LIN7C	-0.2333	0.99859	0.99728	0.9999
ENSG00000149489	rs7124057	-0.0017	0.00085	0.04131	ROM1	0.26464	0.99827	0.9966	0.99993
ENSG00000151240	3	0.00101	0.00042	0.01627	DIP2C	-0.4041	1.00102	1.00019	1.00184
ENSG00000151835	rs17078605	0.00271	0.00095	0.00435	SACS	-0.404	1.00272	1.00085	1.00459
ENSG00000198743	rs8130507	-0.0013	0.00067	0.04941	SLC5A3	-0.4054	0.99869	0.99738	1
ENSG00000153064	8	0.00108	0.00051	0.03626	BANK1	-0.6699	1.00108	1.00007	1.00209
ENSG00000153551	5	0.00075	0.00027	0.00543	CMTM7	0.16542	1.00075	1.00022	1.00128
ENSG00000154122	3	0.00074	0.00028	0.00887	ANKH	-0.3227	1.00074	1.00019	1.0013
ENSG00000155438	2	-0.0008	0.00037	0.02539	NIFK	-0.1542	0.99917	0.99844	0.9999
ENSG00000157514	rs55793580	0.00443	0.00179	0.01339	TSC22D3	-0.2088	1.00444	1.00092	1.00797
ENSG00000157800	4	0.00055	0.00028	0.04904	SLC37A3	0.6047	1.00055	1	1.0011
ENSG00000158882	4	0.00074	0.00031	0.01737	TOMM40	0.39067	1.00074	1.00013	1.00135
ENSG00000159339	6	0.00048	0.00024	0.0424	PADI4	0.3417	1.00048	1.00002	1.00095
ENSG00000159648	rs2241771	-0.0017	0.00056	0.00254	TEPP	0.2146	0.99832	0.99722	0.99941
ENSG00000162910	rs849749	-0.0011	0.00053	0.04478	MRPL55	0.17102	0.99893	0.99788	0.99998
ENSG00000163513	4	0.00084	0.00032	0.00886	TGFBR2	-0.1897	1.00084	1.00021	1.00147
ENSG00000163866	3	0.00076	0.00028	0.007	SMIM12	0.19249	1.00076	1.00021	1.00131
ENSG00000164850	5	0.00072	0.00035	0.0393	GPER1	0.24172	1.00072	1.00004	1.00141
ENSG00000167434	9	0.00059	0.00029	0.04386	CA4	0.73115	1.00059	1.00002	1.00117
ENSG00000168301	rs73087775	-0.0052	0.00148	0.00042	KCTD6	-0.2186	0.9948	0.99193	0.99769
ENSG00000169071	rs4744103	-0.0012	0.00051	0.0147	ROR2	-0.1154	0.99877	0.99778	0.99976
ENSG00000169914	2	0.00134	0.00053	0.01209	OTUD3	-0.2379	1.00134	1.00029	1.00238
ENSG00000172500	5	-0.0009	0.0003	0.00284	FIBP	0.16092	0.99909	0.99849	0.99969
ENSG00000173334	3	-0.0022	0.00095	0.02211	TRIB1	0.25688	0.99782	0.99595	0.99969
ENSG00000174125	3	-0.0008	0.00039	0.04957	TLR1	0.16291	0.99924	0.99849	1
ENSG00000175213	rs9264942	0.02635	0.0017	2.13E-54	ZNF408	0.23314	1.0267	1.02329	1.03012
ENSG00000175806	8	0.00068	0.00026	0.0095	MSRA	0.41119	1.00068	1.00017	1.0012
ENSG00000176170	4	0.00066	0.00027	0.01235	SPHK1	0.49806	1.00066	1.00014	1.00118
ENSG00000179820	6	0.00166	0.00056	0.00288	MYADM	-0.1691	1.00166	1.00057	1.00275
ENSG00000179921	10	-0.0008	0.00031	0.01235	GPBAR1	0.37406	0.99924	0.99864	0.99983
ENSG00000180509	3	0.00057	0.00029	0.04963	KCNE1	0.30337	1.00058	1	1.00115
ENSG00000183696	4	-0.0007	0.00033	0.04621	UPP1	0.40713	0.99934	0.99868	0.99999
ENSG00000183762	5	0.00051	0.00026	0.04907	KREMEN	0.47665	1.00051	1	1.00101
ENSG00000186660	rs7124107	-0.0011	0.00045	0.01006	ZFP91	-0.141	0.99885	0.99798	0.99973
ENSG00000187796	6	0.00042	0.00021	0.04704	CARD9	0.29106	1.00042	1.00001	1.00084
ENSG00000188313	3	0.00061	0.0003	0.04273	PLSCR1	0.44266	1.00061	1.00002	1.0012
ENSG00000188603	2	-0.0014	0.00047	0.00298	CLN3	0.14294	0.99861	0.9977	0.99953
ENSG00000196465	2	-0.0017	0.00075	0.02039	MYL6B	0.32601	0.99825	0.99678	0.99973
ENSG00000198315	rs9366718	-0.0038	0.00187	0.03947	ZKSCAN	-0.1926	0.99616	0.99252	0.99981
ENSG00000059588	4	-0.0023	0.00095	0.01705	TARBP1	-0.3387	0.99773	0.99586	0.99959
ENSG00000198933	4	-0.0009	0.00029	0.00156	TBKBP1	0.33949	0.99908	0.99851	0.99965
ENSG00000203760	rs1591805	0.00217	0.00109	0.04727	CENPW	0.48891	1.00217	1.00003	1.00433
ENSG00000204386	rs3130490	0.00427	0.00112	0.00013	NEU1	0.25124	1.00427	1.00208	1.00647
ENSG00000204421	rs3117572	-0.0086	0.00171	4.33E-07	LY6G6C	0.15657	0.99141	0.9881	0.99473
ENSG00000204444	rs3115663	0.00524	0.00098	8.16E-08	APOM	0.29798	1.00525	1.00333	1.00717
ENSG00000211794	2	-0.0038	0.00134	0.0043	TRAV12-	-0.4417	0.99619	0.99359	0.9988
ENSG00000213516	3	-0.0009	0.00042	0.04004	RBMXL1	-0.2694	0.99914	0.99832	0.99996
ENSG00000214756	rs11322203	-0.0041	0.00159	0.01089	CSKMT	0.16532	0.99596	0.99286	0.99907
ENSG00000225830	rs7098129	0.00164	0.00082	0.04601	ERCC6	-0.2181	1.00164	1.00003	1.00325

ENSG00000231074	3	-0.0019	0.00046	2.81E-05	HCG18	-0.2846	0.99806	0.99716	0.99897
ENSG00000251230	3	0.00099	0.0005	0.04725	MIR3945I	0.67796	1.00099	1.00001	1.00197
ENSG00000260456	rs3748393	-0.0017	0.00079	0.02993	C16orf95	0.34271	0.99829	0.99676	0.99983
ENSG00000264364	rs9903806	0.00206	0.00096	0.03234	DYNLL2	0.26138	1.00206	1.00017	1.00395
