# Supplementary material for: Inflammatory bowel disease activity threatens ankylosing spondylitis: implications from Mendelian randomization combined with transcriptome analysis
Source: Front Immunol. 2024 Feb 28;15:1289049. doi: 10.3389/fimmu.2024.1289049 (PMC10933069; doi:10.3389/fimmu.2024.1289049)
Supplement: Additional File 3 — Detailed information of key DEGs (.pdf). [file DataSheet_3.pdf]

P-values of the correlation analysis between risk gene and immune cell types

Outcome: ukb-a-88

Symbol	CD4+ naive T-cells	CD4+ Tcm	CD8+ T-cells	Mast cells	Memory B-cells	Th1 cells	Th2 cells
PER3	0.317293	0.823399	0.27717	0.384244	0.320718	0.606049	0.372292
TMEM260	0.003771	0.00012	0.005654	0.125938	0.10435	3.72E-05	0.046215
HACE1	0.461608	0.542212	0.22025	0.13543	0.799103	0.000362	4.52E-06
TINF2	0.057716	0.161062	0.195574	0.483723	0.393821	0.148906	0.034862
HNRNPM	0.000126	0.006019	0.000513	0.859224	0.006021	0.023792	0.426684
TRPC4AP	0.005183	0.272248	0.000499	0.598926	0.003576	0.657129	5.84E-05
NPDC1	0.890354	0.027479	0.180188	0.311523	0.157795	3.07E-05	0.599466
GOSR1	0.432465	0.048273	0.191915	0.161721	0.774242	0.000157	0.754816
JHY	0.137119	0.037628	6.53E-05	0.916048	0.03041	0.103732	0.017608
NAA40	0.48877	0.74799	0.673733	0.00521	0.030338	0.347182	0.151444
HPS5	0.327022	0.286397	0.517614	0.546634	0.491466	0.422489	0.119821
USP34	0.701523	0.09342	0.014204	0.125549	0.465726	0.000193	0.096238
SF3B1	0.018872	0.003863	0.001316	0.076193	0.045863	0.986625	0.666793
HPCAL1	0.417286	0.742544	0.003171	0.00086	0.366188	0.475316	2.94E-05
SPOP	0.927453	0.669408	0.017156	0.069064	0.634413	0.115648	0.0137
ACADS	0.265933	0.357152	0.479218	0.782594	0.240591	0.03984	0.000863
HP1BP3	0.000745	0.080295	0.13681	0.215327	0.077135	0.324526	0.561224
IMMT	0.000873	0.011711	0.001532	0.509747	0.006172	0.000536	0.976862
RTP4	0.028062	0.002014	0.00687	0.57998	0.050212	0.475104	0.001274
EPRS1	0.016249	0.575392	0.206716	0.18663	0.127507	0.22373	0.204274
RNF38	0.341543	0.336382	0.134127	0.235453	0.11812	1.64E-05	0.575966
NRM	7.98E-05	0.261431	0.010626	0.751519	0.154388	0.141081	0.017766
ZGRF1	0.023113	0.026788	0.000196	0.971118	0.000615	0.017152	0.467501
SLC16A3	0.004044	0.040222	0.000124	0.62314	0.014771	0.044381	0.043539
PM20D2	0.159856	0.317312	0.83978	0.296508	0.196005	4.20E-08	0.039755
LRMDA	4.95E-06	0.001388	0.002539	0.480588	0.000181	0.698577	0.821816
DIP2C	1.36E-05	4.82E-05	8.85E-08	0.673999	1.61E-05	0.020149	0.977282
SACS	0.002107	0.049336	2.27E-06	0.005293	0.041844	0.562026	0.000655
BANK1	0.068886	0.572071	0.005175	0.14016	3.18E-16	0.466926	0.03134
CMTM7	0.594674	0.057049	0.656839	0.8259	0.409436	0.038173	0.000279
ANKH	0.037702	0.138875	0.660838	0.011813	0.339016	0.039905	0.771564
TSC22D3	0.270957	0.439134	0.666256	0.008344	0.346571	0.816555	0.000525
SLC37A3	0.019611	0.008722	0.025711	0.017244	0.028674	0.000101	0.254601
TOMM40L	0.123522	0.654529	0.997414	0.016562	0.686233	0.053342	0.707206
PADI4	0.000811	0.018559	9.83E-07	0.39292	0.023789	0.004875	0.108271
TGFBR2	0.148443	0.071124	0.035056	0.087386	0.778837	5.01E-06	0.4248
SMIM12	0.038276	0.653595	0.872691	0.224444	0.710468	0.000676	0.634951
GPER1	8.45E-05	0.007409	4.47E-06	0.73883	0.000659	0.027375	0.550639
CA4	0.016048	0.017939	9.18E-06	0.992107	0.004793	0.001572	0.562739
OTUD3	0.013554	0.000569	0.005906	0.16909	0.092874	0.085187	0.820946
ZNF408	0.648277	0.888357	0.411051	0.432364	0.984134	0.00085	0.001765
MSRA	0.018788	0.187786	0.002788	0.801021	0.018918	0.740768	0.006463
SPHK1	0.007198	0.13567	0.015694	0.936288	0.062281	0.109257	0.541069
MYADM	0.254059	0.64673	0.469159	0.060975	0.533505	0.052394	0.08498
KCNE1	1.35E-06	0.000582	1.54E-06	0.803842	0.011849	0.029282	0.606674
KREMEN1	0.051289	6.01E-05	3.02E-08	0.983905	2.20E-05	5.44E-07	0.936493

CARD9	0.000171	0.14192	0.101953	0.802668	0.044379	0.33609	0.047458
PLSCR1	0.00054	4.80E-11	4.62E-10	0.298903	0.000476	7.05E-07	0.025005
CENPW	0.000325	4.83E-05	0.017365	0.190417	0.041084	0.238653	1.04E-06
NEU1	2.31E-06	0.000116	0.000104	0.809823	0.001028	0.134509	0.655962
APOM	0.060367	0.6529	0.04438	0.169903	0.353141	0.61148	0.114757
ERCC6	0.061513	0.295748	0.461411	0.572645	0.322343	0.559598	0.030756
MIR3945HG	0.025248	7.64E-05	1.30E-05	0.752355	0.015606	0.006011	0.43361
DYNLL2	0.969839	0.02658	0.073031	0.453266	0.20451	2.72E-05	0.021093

P-values of the correlation analysis between risk gene and immune cell types

Outcome: finn-b-M13_ANKYLOSPON_STRICT

Symbol	CD4+ naive T-cells	CD4+ Tcm	CD8+ T-cells	Mast cells	Memory B-cells	Th1 cells	Th2 cells
PER3	0.317293	0.823399	0.27717	0.384244	0.320718	0.606049	0.372292
TMEM260	0.003771	0.00012	0.005654	0.125938	0.10435	3.72E-05	0.046215
HACE1	0.461608	0.542212	0.22025	0.13543	0.799103	0.000362	4.52E-06
TINF2	0.057716	0.161062	0.195574	0.483723	0.393821	0.148906	0.034862
HNRNPM	0.000126	0.006019	0.000513	0.859224	0.006021	0.023792	0.426684
TRPC4AP	0.005183	0.272248	0.000499	0.598926	0.003576	0.657129	5.84E-05
NPDC1	0.890354	0.027479	0.180188	0.311523	0.157795	3.07E-05	0.599466
GOSR1	0.432465	0.048273	0.191915	0.161721	0.774242	0.000157	0.754816
JHY	0.137119	0.037628	6.53E-05	0.916048	0.03041	0.103732	0.017608
NAA40	0.48877	0.74799	0.673733	0.00521	0.030338	0.347182	0.151444
HPS5	0.327022	0.286397	0.517614	0.546634	0.491466	0.422489	0.119821
USP34	0.701523	0.09342	0.014204	0.125549	0.465726	0.000193	0.096238
SF3B1	0.018872	0.003863	0.001316	0.076193	0.045863	0.986625	0.666793
HPCAL1	0.417286	0.742544	0.003171	0.00086	0.366188	0.475316	2.94E-05
SPOP	0.927453	0.669408	0.017156	0.069064	0.634413	0.115648	0.0137
ACADS	0.265933	0.357152	0.479218	0.782594	0.240591	0.03984	0.000863
HP1BP3	0.000745	0.080295	0.13681	0.215327	0.077135	0.324526	0.561224
IMMT	0.000873	0.011711	0.001532	0.509747	0.006172	0.000536	0.976862
RTP4	0.028062	0.002014	0.00687	0.57998	0.050212	0.475104	0.001274
EPRS1	0.016249	0.575392	0.206716	0.18663	0.127507	0.22373	0.204274
RNF38	0.341543	0.336382	0.134127	0.235453	0.11812	1.64E-05	0.575966
NRM	7.98E-05	0.261431	0.010626	0.751519	0.154388	0.141081	0.017766
ZGRF1	0.023113	0.026788	0.000196	0.971118	0.000615	0.017152	0.467501
SLC16A3	0.004044	0.040222	0.000124	0.62314	0.014771	0.044381	0.043539
PM20D2	0.159856	0.317312	0.83978	0.296508	0.196005	4.20E-08	0.039755
LRMDA	4.95E-06	0.001388	0.002539	0.480588	0.000181	0.698577	0.821816
DIP2C	1.36E-05	4.82E-05	8.85E-08	0.673999	1.61E-05	0.020149	0.977282
SACS	0.002107	0.049336	2.27E-06	0.005293	0.041844	0.562026	0.000655
BANK1	0.068886	0.572071	0.005175	0.14016	3.18E-16	0.466926	0.03134
CMTM7	0.594674	0.057049	0.656839	0.8259	0.409436	0.038173	0.000279
ANKH	0.037702	0.138875	0.660838	0.011813	0.339016	0.039905	0.771564
TSC22D3	0.270957	0.439134	0.666256	0.008344	0.346571	0.816555	0.000525
SLC37A3	0.019611	0.008722	0.025711	0.017244	0.028674	0.000101	0.254601
TOMM40L	0.123522	0.654529	0.997414	0.016562	0.686233	0.053342	0.707206
PADI4	0.000811	0.018559	9.83E-07	0.39292	0.023789	0.004875	0.108271
TGFBR2	0.148443	0.071124	0.035056	0.087386	0.778837	5.01E-06	0.4248
SMIM12	0.038276	0.653595	0.872691	0.224444	0.710468	0.000676	0.634951
GPER1	8.45E-05	0.007409	4.47E-06	0.73883	0.000659	0.027375	0.550639
CA4	0.016048	0.017939	9.18E-06	0.992107	0.004793	0.001572	0.562739
OTUD3	0.013554	0.000569	0.005906	0.16909	0.092874	0.085187	0.820946
ZNF408	0.648277	0.888357	0.411051	0.432364	0.984134	0.00085	0.001765
MSRA	0.018788	0.187786	0.002788	0.801021	0.018918	0.740768	0.006463
SPHK1	0.007198	0.13567	0.015694	0.936288	0.062281	0.109257	0.541069
MYADM	0.254059	0.64673	0.469159	0.060975	0.533505	0.052394	0.08498
KCNE1	1.35E-06	0.000582	1.54E-06	0.803842	0.011849	0.029282	0.606674
KREMEN1	0.051289	6.01E-05	3.02E-08	0.983905	2.20E-05	5.44E-07	0.936493

CARD9	0.000171	0.14192	0.101953	0.802668	0.044379	0.33609	0.047458
PLSCR1	0.00054	4.80E-11	4.62E-10	0.298903	0.000476	7.05E-07	0.025005
CENPW	0.000325	4.83E-05	0.017365	0.190417	0.041084	0.238653	1.04E-06
NEU1	2.31E-06	0.000116	0.000104	0.809823	0.001028	0.134509	0.655962
APOM	0.060367	0.6529	0.04438	0.169903	0.353141	0.61148	0.114757
ERCC6	0.061513	0.295748	0.461411	0.572645	0.322343	0.559598	0.030756
MIR3945HG	0.025248	7.64E-05	1.30E-05	0.752355	0.015606	0.006011	0.43361
DYNLL2	0.969839	0.02658	0.073031	0.453266	0.20451	2.72E-05	0.021093
