# Supplementary material for: Inflammatory bowel disease activity threatens ankylosing spondylitis: implications from Mendelian randomization combined with transcriptome analysis
Source: Front Immunol. 2024 Feb 28;15:1289049. doi: 10.3389/fimmu.2024.1289049 (PMC10933069; doi:10.3389/fimmu.2024.1289049)
Supplement: Additional File 4 — P-values of the correlation analysis between risk gene and immune cell types (.pdf). [file DataSheet_4.pdf]

MR analysis of pQTL (finn-b-M13_ ANKYLOSPON_STRICT)

outc	exposure	method	nsnp	b	se	pval
AS	10445_20_APOM_ApoM	MR Egger	9	0.6700678	0.3896255	0.1291594
AS	10445_20_APOM_ApoM	Weighted median	9	0.3719909	0.3090485	0.228719
AS	10445_20_APOM_ApoM	IVW	9	0.5018514	0.2285768	0.0281243
AS	10445_20_APOM_ApoM	Simple mode	9	0.4570877	0.4218623	0.3101586
AS	10445_20_APOM_ApoM	Weighted mode	9	0.3778519	0.341963	0.3013022
AS	10638_1_TINF2_TINF2	IVW	2	-0.824195	0.5622764	0.1426978
AS	11130_158_CACNB4_CACB4	MR Egger	3	2.3762781	2.4168448	0.5053879
AS	11130_158_CACNB4_CACB4	Weighted median	3	0.5538981	0.3447578	0.1081356
AS	11130_158_CACNB4_CACB4	IVW	3	0.2078567	0.3028191	0.4924575
AS	11130_158_CACNB4_CACB4	Simple mode	3	0.5634384	0.525907	0.3961455
AS	11130_158_CACNB4_CACB4	Weighted mode	3	0.5735395	0.4240703	0.3088475
AS	11348_132_P4HA2_P4HA2	MR Egger	3	8.3589062	3.0202002	0.2207285
AS	11348_132_P4HA2_P4HA2	Weighted median	3	0.229772	0.6374296	0.7184983
AS	11348_132_P4HA2_P4HA2	IVW	3	0.2514812	1.2132632	0.8357938
AS	11348_132_P4HA2_P4HA2	Simple mode	3	0.970751	1.3002383	0.5331415
AS	11348_132_P4HA2_P4HA2	Weighted mode	3	0.4121724	0.7724804	0.6469981
AS	13022_20_TP53I11_P5I11	MR Egger	16	0.3520067	0.1859572	0.0792236
AS	13022_20_TP53I11_P5I11	Weighted median	16	0.3885566	0.1458686	0.0077277
AS	13022_20_TP53I11_P5I11	IVW	16	0.2695905	0.109962	0.0142195
AS	13022_20_TP53I11_P5I11	Simple mode	16	0.3787406	0.2999079	0.2259198
AS	13022_20_TP53I11_P5I11	Weighted mode	16	0.3630734	0.1471856	0.0261642
AS	18337_4_GMDS_GMDS	Wald ratio	1	0.4860627	0.8908663	0.5853363
AS	3186_2_C2_C2	MR Egger	20	0.242694	0.1590372	0.1443843
AS	3186_2_C2_C2	Weighted median	20	0.1812185	0.1441355	0.2086529
AS	3186_2_C2_C2	IVW	20	0.197181	0.1156318	0.0881481
AS	3186_2_C2_C2	Simple mode	20	0.0682907	0.2575429	0.7937398
AS	3186_2_C2_C2	Weighted mode	20	0.2068502	0.1432049	0.164906
AS	3346_72_AURKB_AURKB	MR Egger	8	-0.101313	0.4375429	0.8245824
AS	3346_72_AURKB_AURKB	Weighted median	8	-0.089887	0.1641419	0.5839562
AS	3346_72_AURKB_AURKB	IVW	8	-0.009185	0.1658636	0.9558388
AS	3346_72_AURKB_AURKB	Simple mode	8	-0.173359	0.3764503	0.6591155
AS	3346_72_AURKB_AURKB	Weighted mode	8	-0.117874	0.1674304	0.5041612
AS	5006_71_MAPK13_MK13	IVW	2	-0.333926	0.2913681	0.2517689
AS	5007_1_MAPK14_MAPK14	Wald ratio	1	-0.562665	0.8488579	0.5074267
AS	6525_17_DUSP13_DUSP13	MR Egger	5	-0.104399	0.5381899	0.858582
AS	6525_17_DUSP13_DUSP13	Weighted median	5	-0.364927	0.344328	0.2892249
AS	6525_17_DUSP13_DUSP13	IVW	5	-0.373279	0.2755771	0.1755661
AS	6525_17_DUSP13_DUSP13	Simple mode	5	-0.356692	0.5049289	0.5189019
AS	6525_17_DUSP13_DUSP13	Weighted mode	5	-0.306778	0.434312	0.5189386
AS	6536_54_PLBD2_LAML2	MR Egger	3	0.9583057	2.3728911	0.7556489
AS	6536_54_PLBD2_LAML2	Weighted median	3	-0.107765	0.2872313	0.7075214
AS	6536_54_PLBD2_LAML2	IVW	3	-0.067951	0.4938834	0.8905681
AS	6536_54_PLBD2_LAML2	Simple mode	3	-0.392114	0.5124376	0.5241206
AS	6536_54_PLBD2_LAML2	Weighted mode	3	-0.174949	0.3312866	0.6501786
AS	8356_88_OXT_NEU1	MR Egger	5	0.2345239	0.3923863	0.5921582
AS	8356_88_OXT_NEU1	Weighted median	5	0.1045064	0.1377424	0.4480267
AS	8356_88_OXT_NEU1	IVW	5	0.093348	0.2232246	0.6758155
AS	8356_88_OXT_NEU1	Simple mode	5	0.2255768	0.511209	0.6818266
AS	8356_88_OXT_NEU1	Weighted mode	5	0.0825028	0.1307795	0.5623848

AS	8982_65_THBS3_TSP3	MR Egger	12	-0.799322	0.5264423	0.1598884
AS	8982_65_THBS3_TSP3	Weighted median	12	-0.320507	0.2599344	0.217565
AS	8982_65_THBS3_TSP3	IVW	12	-0.382353	0.2242424	0.088178
AS	8982_65_THBS3_TSP3	Simple mode	12	-0.075898	0.3594667	0.8366389
AS	8982_65_THBS3_TSP3	Weighted mode	12	-0.281203	0.3187985	0.3966042
AS	9504_19_RAB27A_RB27A	IVW	2	-0.092675	0.3997478	0.8166666

MR analysis of pQTL (ukb-a-88)

outcome	exposure	method	nsnp	b	se	pval
UKB-a-88	10424_31_NPDC1_NPDC1	MR Egger	3	-0.02339	0.024981	0.5209
UKB-a-88	10424_31_NPDC1_NPDC1	Weighted median	3	-0.00082	0.001909	0.667842
UKB-a-88	10424_31_NPDC1_NPDC1	IVW	3	-0.00074	0.003738	0.843804
UKB-a-88	10424_31_NPDC1_NPDC1	Simple mode	3	0.001357	0.003869	0.759313
UKB-a-88	10424_31_NPDC1_NPDC1	Weighted mode	3	-0.00056	0.002636	0.850701
UKB-a-88	10445_20_APOM_ApoM	MR Egger	10	-0.00134	0.000977	0.208948
UKB-a-88	10445_20_APOM_ApoM	Weighted median	10	-0.00131	0.000694	0.060077
UKB-a-88	10445_20_APOM_ApoM	IVW	10	-0.00112	0.000649	0.083524
UKB-a-88	10445_20_APOM_ApoM	Simple mode	10	0.000668	0.001308	0.621864
UKB-a-88	10445_20_APOM_ApoM	Weighted mode	10	-0.00128	0.00072	0.108371
UKB-a-88	10638_1_TINF2_TINF2	IVW	2	4.51E-05	0.001769	0.979673
UKB-a-88	11405_150_CARD9_CARD9	MR Egger	4	0.000559	0.005768	0.931674
UKB-a-88	11405_150_CARD9_CARD9	Weighted median	4	0.000599	0.000826	0.468163
UKB-a-88	11405_150_CARD9_CARD9	IVW	4	0.001306	0.001257	0.29874
UKB-a-88	11405_150_CARD9_CARD9	Simple mode	4	0.000532	0.001605	0.762204
UKB-a-88	11405_150_CARD9_CARD9	Weighted mode	4	0.000309	0.000876	0.74796
UKB-a-88	11493_169_DYNLL2_DYL2	Wald ratio	1	0.005002	0.002461	0.042065
UKB-a-88	12783_29_HNRNPM_HNRPM	MR Egger	3	0.002391	0.001694	0.392519
UKB-a-88	12783_29_HNRNPM_HNRPM	Weighted median	3	0.000171	0.000804	0.831239
UKB-a-88	12783_29_HNRNPM_HNRPM	IVW	3	6.63E-05	0.000958	0.944842
UKB-a-88	12783_29_HNRNPM_HNRPM	Simple mode	3	-0.00223	0.001982	0.376894
UKB-a-88	12783_29_HNRNPM_HNRPM	Weighted mode	3	0.000646	0.00079	0.499576
UKB-a-88	13727_44_SPOP_SPOP	IVW	2	0.000988	0.001111	0.374025
UKB-a-88	14069_61_CA4_Carbonic_Anhydra	MR Egger	7	-0.00075	0.000705	0.335502
UKB-a-88	14069_61_CA4_Carbonic_Anhydra	Weighted median	7	-0.00028	0.00059	0.639355
UKB-a-88	14069_61_CA4_Carbonic_Anhydra	IVW	7	-0.00031	0.000515	0.541666
UKB-a-88	14069_61_CA4_Carbonic_Anhydra	Simple mode	7	0.000388	0.001159	0.749205
UKB-a-88	14069_61_CA4_Carbonic_Anhydra	Weighted mode	7	-0.00026	0.000632	0.700536
UKB-a-88	17331_138_KREMEN1_KREM1	MR Egger	19	-0.00058	0.000704	0.422136
UKB-a-88	17331_138_KREMEN1_KREM1	Weighted median	19	0.000767	0.000612	0.210122
UKB-a-88	17331_138_KREMEN1_KREM1	IVW	19	0.000355	0.000419	0.397317
UKB-a-88	17331_138_KREMEN1_KREM1	Simple mode	19	0.000132	0.001147	0.909652
UKB-a-88	17331_138_KREMEN1_KREM1	Weighted mode	19	0.00059	0.000769	0.452777
UKB-a-88	17393_13_ACADS_SCAD	MR Egger	8	0.00053	0.000974	0.60563
UKB-a-88	17393_13_ACADS_SCAD	Weighted median	8	0.000285	0.000526	0.588255
UKB-a-88	17393_13_ACADS_SCAD	IVW	8	0.0003	0.000453	0.507539
UKB-a-88	17393_13_ACADS_SCAD	Simple mode	8	0.000435	0.000836	0.618409
UKB-a-88	17393_13_ACADS_SCAD	Weighted mode	8	0.00024	0.000581	0.691602
UKB-a-88	18315_38_RTP4_RTP4	MR Egger	6	0.000723	0.00076	0.395433
UKB-a-88	18315_38_RTP4_RTP4	Weighted median	6	0.000503	0.000468	0.282475
UKB-a-88	18315_38_RTP4_RTP4	IVW	6	0.000394	0.000439	0.369967
UKB-a-88	18315_38_RTP4_RTP4	Simple mode	6	0.000171	0.000977	0.867591
UKB-a-88	18315_38_RTP4_RTP4	Weighted mode	6	0.000509	0.000493	0.348932
UKB-a-88	4294_16_SPHK1_Sphingosine_kin	MR Egger	6	0.001177	0.001642	0.513111
UKB-a-88	4294_16_SPHK1_Sphingosine_kin	Weighted median	6	-0.00019	0.001176	0.868841
UKB-a-88	4294_16_SPHK1_Sphingosine_kin	IVW	6	0.001184	0.001041	0.255458
UKB-a-88	4294_16_SPHK1_Sphingosine_kin	Simple mode	6	-0.00059	0.00194	0.772099
UKB-a-88	4294_16_SPHK1_Sphingosine_kin	Weighted mode	6	-0.0007	0.001909	0.729657
UKB-a-88	5133_17_TGFBR2_TGF_b_R_II	MR Egger	3	-0.00043	0.002109	0.870755

UKB-a-88	5133_17_TGFBR2_TGF_b_R_II	Weighted median	3	0.000728	0.000877	0.406234
UKB-a-88	5133_17_TGFBR2_TGF_b_R_II	IVW	3	0.000871	0.000833	0.29557
UKB-a-88	5133_17_TGFBR2_TGF_b_R_II	Simple mode	3	0.001458	0.001637	0.466958
UKB-a-88	5133_17_TGFBR2_TGF_b_R_II	Weighted mode	3	0.000532	0.000891	0.610811
UKB-a-88	7805_52_GOSR1_GOS_28	MR Egger	7	0.001856	0.001685	0.32091
UKB-a-88	7805_52_GOSR1_GOS_28	Weighted median	7	0.000417	0.00068	0.540117
UKB-a-88	7805_52_GOSR1_GOS_28	IVW	7	0.001142	0.000698	0.101777
UKB-a-88	7805_52_GOSR1_GOS_28	Simple mode	7	0.000305	0.001273	0.818843
UKB-a-88	7805_52_GOSR1_GOS_28	Weighted mode	7	0.000519	0.00067	0.468369
UKB-a-88	8356_88_OXT_NEU1	MR Egger	6	0.00037	0.000664	0.6074
UKB-a-88	8356_88_OXT_NEU1	Weighted median	6	0.000108	0.000426	0.800004
UKB-a-88	8356_88_OXT_NEU1	IVW	6	0.00015	0.000395	0.704365
UKB-a-88	8356_88_OXT_NEU1	Simple mode	6	-0.00017	0.000772	0.83506
UKB-a-88	8356_88_OXT_NEU1	Weighted mode	6	0.000151	0.000413	0.729922
UKB-a-88	8864_59_CENPW_CENPW	IVW	2	0.002161	0.00239	0.365816
UKB-a-88	9049_2_SLC16A3_MOT4	Wald ratio	1	0.002164	0.002904	0.456223
