# Supplementary material for: Inflammatory bowel disease activity threatens ankylosing spondylitis: implications from Mendelian randomization combined with transcriptome analysis
Source: Front Immunol. 2024 Feb 28;15:1289049. doi: 10.3389/fimmu.2024.1289049 (PMC10933069; doi:10.3389/fimmu.2024.1289049)
Supplement: Additional File 5 — Detailed information of MR analysis of pQTL (.pdf). [file Table_1.docx]

**Table S1 Datasets Information**

| ID | Sample | Case | Control | Number of SNPs | Year | Type |
| --- | --- | --- | --- | --- | --- | --- |
| ieu-a-31 | 34652 | 12,882 | 21,770 | 12,716,084 | 2015 | IBD |
| ebi-a-GCST004131 | 59,957 | 25,042 | 34,915 | 9,619,016 | 2017 | IBD |
| finn-b-K11_IBD_STRICT | 214053 | 3,753 | 210,300 | 16,380,455 | 2021 | IBD |
| ukb-a-88 | 337,159 | 968 | 336,191 | 10,894,596 | 2017 | AS |
| finn-b-M13_ANKYLOSPON_STRICT | 218,030 | 599 | 217,431 | 16,380,466 | 2021 | AS |
